# β‐elemene promotes ferroptosis to improve the sensitivity of imatinib in gastrointestinal stromal tumours by targeting N6AMT1

**DOI:** 10.1002/ctm2.70438

**Published:** 2025-08-27

**Authors:** Jin Lan, Weili Zhang, Kaixuan Zeng, Cong Li, Jiahua He, Xinyue Li, Rong Yang, Jun Chi, Zhigang Hong, Weifeng Wang, Chi Zhou, Binyi Xiao, Wenhua Fan, Junzhong Lin, Qingjian Ou, Yujing Fang, Zhizhong Pan, Jianhong Peng, Weihao Li, Xiaojun Wu

**Affiliations:** ^1^ State Key Laboratory of Oncology in South China Collaborative Innovation Center for Cancer Medicine Sun Yat‐sen University Cancer Center Guangzhou China; ^2^ Department of Colorectal Surgery Sun Yat‐sen University Cancer Center Guangzhou China; ^3^ Precision Medical Research Institute The Second Affiliated Hospital of Xi'an Jiaotong University Xi'an China; ^4^ Department of Intensive Care Unit Sun Yat‐sen University Cancer Center Guangzhou China; ^5^ Department of Endoscopy Sun Yat‐sen University Cancer Center Guangzhou China

**Keywords:** ferroptosis, gastrointestinal stromal tumours, imatinib resistance, ROS, β‐elemene

## Abstract

**Background:**

Imatinib has been widely used in gastrointestinal stromal tumours and significantly improved the prognosis of GIST patients, but approximately half of patients develop acquired treatment resistance, highlighting the urgency for novel therapeutic strategies.

**Methods:**

A variety of bioinformatic tools and laboratory experiments, RNA sequencing, animal models and the thermal proteome profiling assay were employed to validate our findings and investigate the antitumour effects of β‐elemene.

**Results:**

We found that imatinib‐resistant GIST was associated with negative regulation of ferroptosis activity, and inducing ferroptosis can enhance the sensitivity of resistant cells to imatinib. Furthermore, we found that β‐elemene enhances imatinib sensitivity in imatinib‐resistant GIST cells through inducing ferroptosis. Moreover, the combination treatment of β‐elemene and imatinib showed significantly increased antitumour efficacy, compared to each monotherapy, both in vitro and in vivo. Mechanistically, β‐elemene specifically targets N6AMT1, inhibiting its transcriptional repression function and activating the nuclear factor erythroid 2‐related factor 2 (NRF2)‐HMOX1 signalling pathway to induce ferroptosis.

**Conclusion:**

Β‐elemene can target N6AMT1 and promote ferroptosis by increasing the expression of NRF2 and HMOX1. These findings suggest β‐elemene as a prospective therapeutic strategy to improve the sensitivity of imatinib in gastrointestinal stromal tumours.

**Key points:**

- l Imatinib resistance is associated with ferroptosis activity in GIST.
- l Combination of β‐elemene and imatinib effectively treats gastrointestinal stromal tumours both in vivo and in vitro.
- l β‐elemene promotes imatinib sensitivity in GIST through ferroptosis.
- l N6AMT1 is a potential target of β‐elemene.
- l β‐elemene targets N6AMT1 to promote imatinib sensitivity in imatinib‐resistant GIST cells via the NRF2/HMOX1 axis.

## Graphical abstract

β‐elemene specifically targets N6AMT1, inhibiting its transcriptional repression function and activating the nuclear factor erythroid 2‐related factor 2‐HMOX1 signalling pathway to induce ferroptosis.

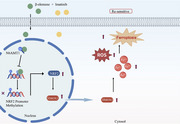

## 1 INTRODUCTION

Gastrointestinal stromal tumours (GISTs) are the most common gastrointestinal mesenchymal malignancy of the gastrointestinal tract, with an estimated incidence of 10–20 per million population.^1^, ^2^ GISTs are mainly caused by mutations in KIT or PDGFRA.^3^ Imatinib is a tyrosine kinase inhibitor targeting BCR‐ABL, KIT and PDGFR, which has a highly inhibitory effect on GISTs and has been approved as the first‐line treatment for KIT‐positive GISTs.^4^ However, about 50% of patients developed drug resistance after 2 years of treatment with imatinib.^5^ Secondary mutations partly explained the resistance of imatinib in GISTs with secondary imatinib resistance,^6^, ^7^ while approximately 40% of patients with no secondary mutations.^8^, ^9^ There are limited treatment options available for those patients without secondary mutations. The overall efficacy of these therapies is poor. Therefore, analysing the molecular mechanism of imatinib resistance is extremely urgent and must be characterised to improve the long‐term prognosis of GIST patients by devising novel therapies.

Resisting cell death is one of the most important characteristics of cancer cells, and it is also the main reason for drug resistance.^10^ Ferroptosis is a non‐apoptotic form of cell death characterised by the accumulation of iron and lipid peroxidation, which has become an effective target of anti‐cancer therapy.^11^ Recent evidence suggests that ferroptosis is related to cancer chemotherapy resistance, and induction of ferroptosis can reverse drug resistance.^12^, ^13^ Ferroptosis can be induced by inhibiting ADP ribosylation factor 6 (ARF6) to activate active fatty acid synthase 4(ACSL4), thereby overcoming gemcitabine resistance in pancreatic cancer.^14^ In addition, the PARP inhibitor olaparib is used in combination with ferroptosis inducers to enhance the sensitivity of BRCAproficient ovarian cancer cells to olaparib by inhibiting solute carrier family 7 member 11 (SLC7A11)‐mediated GSH synthesis.^15^ These results indicate the possibility of utilising ferroptosis as a strategy to overcome therapeutic resistance.

β‐elemene, a bioactive compound isolated from the Chinese herb Curcumae Rhizoma, exhibits a low‐toxicity and broad‐spectrum anticancer effect and is used in various cancer types.^16^ Several studies have revealed that β‐elemene plays an antitumour role through different pathways, such as activating mitochondrial‐dependent pathways, inducing apoptosis or arresting the cell cycle.^17^ More interestingly, β‐elemene is used clinically for radiation sensitisation and chemotherapy of various tumours, and it can effectively reverse drug resistance.^18^, ^19^ Previous studies reported that β‐elemene produces combinative effects with some chemotherapies, such as oxaliplatin and gefitinib.^20^, ^21^ It was reported that β‐elemene could induce ferroptosis in lung cancer and colorectal cancer (CRC). The combined therapy of β‐elemene and cetuximab is sensitive to KRAS mutant CRC cells by inducing ferroptosis and inhibiting EMT.^22^ However, the role of β‐elemene in imatinib‐resistant GIST cells and the targets for β‐elemene are not fully illustrated. Therefore, systematic exploration of the combined application of β‐elemene with imatinib to achieve better therapeutic effects is of great clinical significance given the grim reality of GIST treatment.

In this study, we aimed to investigate the role of ferroptosis in regulating imatinib resistance in GIST cells. The results showed that inducing ferroptosis enhanced the sensitivity of resistant cells to imatinib. Furthermore, we found that combinative treatment with imatinib and β‐elemene can hyperactivate HMOX1 and eventually lead to ferroptosis. Moreover, by thermal proteome profiling (TPP), we identified N6AMT1 as the target protein of β‐elemene. Mechanistically, β‐elemene deactivates N6AMT1 and then induces ferroptosis via the nuclear factor erythroid 2‐related factor 2 (NRF2)‐HMOX1 signalling axis. Taken together, our work therefore provides a new therapeutic strategy for imatinib resistance in patients with advanced GIST.

## 2 MATERIALS AND METHODS

### 2.1 Cell culture and reagents

The GIST cell line GIST‐882 was purchased from the American Type Culture Collection (ATCC, Manassas, VA, USA). GIST‐T1 was a gift from Professor Haibo Qiu (Sun Yat‐sen University Cancer Center [SYSUCC]), cultured as described in previous literature. GIST‐T1and GIST‐882 cells were cultured in RPMI1640 medium supplemented with 15% fetal bovine serum, 1% penicillin/streptomycin, 1% l‐glutamine and 2 µg/mL Gentamycin.

To establish cells resistant to imatinib, GIST‐882 and GIST‐T1 cells were exposed to gradually increasing concentrations of imatinib as previously described. GIST cell lines were generated through at least 6 months of drug resistance. The administration of the specific imatinib concentration was continued until the cells grew normally. Subsequently, the concentration was increased, and the above process was repeated to obtain imatinib‐resistant cell lines through repeated induction. The cell counting kit‐8 (CCK‐8) assay was used to evaluate the half‐maximal inhibitory concentration (IC50) of GIST cell lines. For the functional assays, imatinib was removed for at least 1 week to avoid acute effects. All cell lines were cultured in an incubator with 37 ° C and 5% CO_2_.

β‐elemene was provided by Dalian HolleyKingkong Pharmaceutical Co. Ltd. Imatinib (T6230) was purchased from TargetMol. Ferroptosis activators erastin (HY‐15763) and RAS‐selective lethal 3 (RSL3; HY‐100218) were purchased from MedChemExpress. Ferroptosis inhibitor ferrostatin‐1 (Fer‐1, HY‐100579) was purchased from MedChemExpress. Hemin (51280) and zinc protoporphyrin‐9 (ZnPP, MB4231) were purchased from Sigma‐Aldrich and Meilunbio, respectively.

### 2.2 Cell viability assay

Cell viability was evaluated using the CCK‐8 (LJ621, Dojindo) according to the product manual. A total of 2000 GIST cells were seeded in 96‐well plates and treated with corresponding processes. After the cells were treated with drugs, the medium was replaced with 100 µL fresh medium containing 10 µL CCK‐8 reagent. After incubation for 2 h, the absorbance was detected at a wavelength of 450 nm, and growth curves were generated to determine the cell inhibition rate.

To calculate IC50 values for all drugs, GraphPad Prism Software was used. We utilised the CompuSyn software (ComboSyn, Inc.) to assess the drug synergism effect, which involves the computation of the combination index (CI). CI values offer quantitative definitions for additive effect (CI = 1), synergism (CI < 1), and antagonism (CI > 1).

### 2.3 Cell death assay

Cell death was measured by propidium iodide (PI; SIGMA, Cat#537059) staining. Cells were seeded in six‐well plates 1 day before treatment with drugs. On the next day, cells were treated with the indicated drugs for 48 h, all cells including the floating cells in the culture medium were harvested by trypsinisation and resuspended in fresh PBS containing PI. After incubation for 10 min, the cells were analysed by flow cytometry. Data were analysed using FlowJo software.

### 2.4 Colony formation assay

Cells were seeded in six‐well plates (2000 cells per well) with three replicates. Following cell adhesion, the medium was replaced with a medium containing the indicated drugs. After 14 days with a complete medium at 37°C with 5% CO_2_. Cells were then fixed with methanol and stained with crystal violet for 30 min at 25°C, washed with ddH_2_O and the colonies of each well were counted.

### 2.5 Western blot analysis

Cells and tissues were lysed in a lysis buffer (50 mM TrisHCl, pH 7.4, 150 mM NaCl, 1% NP‐40 [Beyotime, P0013F], .1% SDS, .5% sodium deoxycholate [Sigma‐Aldrich, 302‐954], 1 mM EDTA and 10% glycerol) and centrifuged at 13 000 × g for 30 min at 4°C. Protein concentration was determined using a BCA Protein Assay Kit (Thermo Fisher Scientific). Equal amounts of total protein lysates were separated on 10% SDS‐PAGE and transferred to a PVDF membrane (Bio‐Rad). Subsequently, the membranes were blocked with 5% non‐fat milk at room temperature for 1 h, followed by an overnight incubation at 4°C with primary antibodies. Primary antibodies used in this study are anti‐caspase‐3 (Proteintech, 19677‐1‐AP), anti‐cleaved caspase‐3 (CST, 9664S), anti‐caspase‐7 (Proteintech, 27155‐1‐AP), anti‐cleaved caspase‐7 (CST, 9491T), anti‐GSDMD (Proteintech, 20770‐1‐AP), anti‐GSDME (Proteintech, 13075‐1‐AP), anti‐glutathione peroxidase 4 (GPX4; Affinity Biosciences, DF6701), anti‐ferritin heavy chain (FTH1; Affinity Biosciences, DF4828), anti‐ACSL3 (Affinity Biosciences, DF9606), anti‐vinculin (CST, 13901S), anti‐HMOX1 (Proteintech, 10701‐1‐AP), anti‐NRF2 (Proteintech, 16396‐1‐AP), anti‐N6AMT1 (Proteintech, 16211‐1‐AP), anti‐SLC7A11 (Proteintech, 26864‐1‐AP), anti‐FSP1 (Proteintech, 20886‐1‐AP), anti‐dihydroorotate dehydrogenase (DHODH; Proteintech, 14877‐1‐AP) and anti‐SMYD2 (Proteintech, 21290‐1‐AP). The PVDF membrane was washed with TBST buffer and incubated with the secondary antibodies labelled with HRP‐conjugated secondary antibodies (Neobioscience) for 2 h at room temperature. Protein signals were detected with an enhanced chemiluminescent kit (Thermo Scientific).

### 2.6 Human specimens and immunohistochemistry (IHC) analysis

Paraffin‐embedded GIST samples were collected from 40 patients who underwent surgical therapy at SYSUCC. Written consent was obtained from the participating patients before surgery, and the clinical and histopathological data provided to researchers were anonymised. The study was approved by the Institutional Research Ethics Committee of SYSUCC (Guangzhou, China, Approval Number: B2022‐465‐01).

For IHC staining, paraffin‐embedded GIST specimens were performed as described previously. Primary antibodies used in this study are anti‐GPX4 (Affinity Biosciences, DF6701), anti‐FTH1 (Affinity Biosciences, DF4828), anti‐Ki 67 (Abcam, ab15580), anti‐HMOX1 (Proteintech, 10701‐1‐AP), anti‐NRF2 (Proteintech, 16396‐1‐AP), anti‐4‐hydroxynonenal (R&D, MAB3249). IHC scores were recorded as the degree of staining intensity and percentage of positive cells. The staining extent that scored according to the percentage of positively stained cells ranged from 0 to 3 (0, 0%–5%; 1, 5%–25%; 2, 26%–50%; 3, 51%–75%; and 4, 76%–100%), although the intensity of staining was scored as 0 (negative staining), 1 (weak staining), 2 (moderate staining), and 3 (strong staining). Final IHC staining scores were evaluated by two independent gastrointestinal pathologists blinded to the patient's clinical characteristics.

### 2.7 Detection of intracellular Fe^2+^

Cells were inoculated in culture dishes with a cell density of 1 × 10^4^/mL and treated according to designated groups for 24 h. The cells were incubated with 1 µmol/L FerroOrange working solution (Dojindo) for 30 min and then observed under a fluorescence microscope. All images were acquired with the same instrument parameters and processed with the same settings to maximise the ability to compare results between conditions.

### 2.8 Malondialdehyde (MDA)‐level measurement

Cells were seeded at a density of 1 × 10^7^cells per 60 mm culture dish and treated according to designated groups. The relative concentration of MDA was measured using the MDA assay kit (Dojindo) according to the manufacturer's instructions. Protein concentration was assayed using a Thermo Fisher Scientific BCA Protein Assay Kit according to the manufacturer's instructions.

### 2.9 Assessment of total cellular reactive oxygen species (ROS)

Dichlorodihydrofluorescein diacetate (DCFH‐DA) assay kit (Beyotime) was used to detect intracellular ROS production. Cells were seeded in six‐well plates and treated with the indicated drugs.

Then, cells were incubated with DCFH–DA at a final concentration of 10 µM in medium without FBS at 37°C for 30 min and washed three times with medium. The level of ROS was determined by flow cytometer. The level of ROS generation was analysed with FlowJo software (FlowJo).

### 2.10 Lipid peroxidation detection

Lipid peroxidation was detected using C11 BODIPY581/591 (Thermo Fisher Scientific). GIST cells (1 × 10^4^ cells per dish) were plated in culture dishes and incubated for the indicated drugs for 24 h. After treatment, the cells were incubated in a medium containing 5 µM BODIPY 581/591 C11 at 37°C for 30 min for live‐cell imaging. The cells were imaged at 40 × magnification using a Leica microscope to detect the oxidised forms of the probe. All images were acquired with the same instrument parameters and processed with the same settings to maximise the ability to compare results between conditions.

### 2.11 Transmission electron microscopy (TEM) examination

GIST cells were seeded in dishes and subjected to different treatments. After 24 h, cells were incubated with a pre‐chilled 2% glutaraldehyde solution for 2 h at 4°C to fix the cell pellet. The cells were stained with 2% uranyl acetate solution for 2 h and then dehydrated in 50%, 70%, 90%, 95% and 100% acetone. The cells were embedded in Spurr embedding kit (KYD bio, 14300), and ultrathin sections were prepared for observation under an electron microscope (HITACHI).

### 2.12 Cell transfection and lentivirus production

The small interfering RNA (siRNA) targeting N6AMT1 and SMYD2 were synthesised by GENE CREATE. Transfection was conducted using jetPRIME reagent (Polyplus, 101000046) as recommended. The siRNA sequences used for gene knockdown in this study were listed in Supporting Information Table S1. After transfection for 8 h, the medium was refreshed with complete medium, and these cells were cultivated overnight in an incubator before being re‐seeded for experiments.

For overexpression of HMOX1 and N6AMT1, negative control or over‐expressing plasmids were co‐transfected into HEK293T with pHelper and pEnv. The virus was harvested from the transfected HEK293T cells, and the GIST cells were then infected with the viral supernatants for 2 consecutive days. Stable GIST cell lines were selected by treating with puromycin (2 µg/mL) for 10 days.

### 2.13 Quantitative real‐time polymerase chain reaction (RT‐qPCR)

The total RNA was extracted using TRIzol reagent (Invitrogen, 15596026) and adjusted to 200 µg/mL. .5 µg total RNA was used for reverse transcription. Complementary DNA was generated using the PrimeScript RT reagent kit (TaKaRa Bio, RR037A) according to the manufacturer's instructions. Finally, the designed primers (Table S2) were used for RT‐qPCR in the LightCycler 480 (Roche Diagnostics) thermal cycler, and each sample was analysed in triplicate.

### 2.14 RNA sequencing (RNA‐seq) and analysis

RNA‐seq was performed by BGI as described before. In brief, after GIST‐T1‐IR cells were treated with DMSO or β‐elemene for 12 h, the total RNA was extracted using TRIzol reagent (Invitrogen, 15596026). RNA‐seq and the following bioinformatic analyses were performed using the Illumina HiSeq platform (Illumina) as previously described. Analysis and visualisation of the differentially expressed genes (DEGs) were conducted using the limma and ggplot 2 packages in R software. The Kyoto Encyclopedia of Genes and Genomes (KEGG) analysis was used for gene functional annotation. We established the criteria as the *p*‐value < .05 and log2 fold change > .5.

### 2.15 TPP experiment

TPP is a novel target screening method that can predict direct targets for small drug molecules. The TPP principle uses the changes in the thermodynamic properties of protein stability that occur when ligands bind to small molecules by subjecting intact cells or protein lysates to a temperature gradient in the presence of small molecules to precipitate insoluble heat‐denatured proteins. The soluble fraction of the protein was then recovered and quantified by LC‐mass spectrometry. TPP was performed according to previous reports.^23^

### 2.16 Cellular thermal shift assay (CETSA)

The CETSA was utilised to evaluate the stability of the target protein following the previous protocol. GIST‐882‐IR and GIST‐T1‐IR cells were lysed by repeated freezing and thawing of the suspension in liquid nitrogen three times and centrifuging at 4°C. The supernatant was collected and treated with Dimethyl sulfoxide(DMSO) or β‐elemene (20 µg/L) for 1 h at 25°C. The mixtures were then divided into several tubes and heated at various temperatures ranging from 37 to 67°C for 3 min, cooled at room temperature for 3 min and then centrifuged at 4°C. Loading buffer was added to the samples, followed by boiling at 98°C for 10 min. The samples were subsequently analysed using Western blotting analysis.

### 2.17 Molecular docking

The crystal structure of N6AMT1 used for docking was obtained from the RCSB Protein Data Bank website (https://www.rcsb.org). Modifications, including the elimination and hydrogenation of ethanol and water molecules, as well as the refinement of amino acids, were performed using PyMol 2.5.42.^24^ The 3D chemical structure of β‐elemene was obtained from PubChem (https://pubchem.ncbi.nlm.nih.gov). AutoDock 1.5.7 software was adopted for molecular docking.^25^ For docking analysis, all protein and molecular files were converted into PDBQT format. The grid box was centred to cover the domain of each protein and to accommodate free molecular movement. The grid box was set to 30 × 30 × 30 Å, and the grid point distance was .05 nm. The output docking conformation with the highest scoring was considered by us as the bound conformation, and finally PyMol 2.5.4 was used for visualisation.

### 2.18 Synthesis of biotin‐labelled Β‐elemene and pull‐down assay

β‐elemene and biotin‐EDA(Biotin‐Ethylenediamine) were dissolved in N,N‐Dimethylformamide(DMF), followed by the addition of EDCI, HOBT and DMAP. The reaction was conducted at room temperature (RT) for 15 h. Subsequently, purification was performed via HPLC (acetonitrile: water = 65%∼95%, .1% TFA, flow rate 20 mL/min, RT) to obtain β‐elemene‐biotin. To evaluate the binding effect between β‐elemene and β‐elemene‐biotin for N6AMT1, the GIST cell lysates were preincubated with β‐elemene for 1 h, then incubated with 20 µM β‐elemene‐biotin for 1 hat 4°C. The β‐elemene‐biotin complex was then captured using streptavidin magnetic beads for 1 h at 4°C, washed three times and finally analysed by western blot.

### 2.19 Bioinformatic analysis

The GIST gene expression profiles GSE132542 was obtained from the publicly available Gene Expression Omnibus database (http://www.ncbi.nlm.nih.gov/geo/). The GSE132542 dataset contained 29 samples, including 14 imatinib‐naïve and 15 imatinib‐resistant GISTs. We then used the web‐based visualisation and inference toolbox(eVITTA, easy Visualization and Inference Toolbox for Transcriptome Analysis, https://tau.cmmt.ubc.ca/eVITTAL) for transcriptome and Gene set enrichment analysis (GSEA)^26^. Enrichment results were considered significant where the *p*‐value < .05 and FDR  < .25.

### 2.20 Immunofluorescence staining

The cells were inoculated in petri dishes covered with a coverslip, treated according to the groups and collected, rinsed and fixed with 4% paraformaldehyde for 15 min, permeated with .5% Triton X‐100 for 20 min and sealed with goat serum for 30 min. Then the diluted primary antibody (NRF2, Proteintech, 16396‐1‐AP) was added, and the cells were incubated at 4°C overnight. After the washing with PBST three times, Cy3‐labelled Sheep Anti‐Rabbit Fluorescent Secondary Antibody IgG (Wuhan Bode Bioengineering Co. Ltd., BA1032) was added and the cells were incubated at room temperature for 1 h in darkness. After washing with PBST three times, the cells were stained with DAPI (Beyotime, C1002) and sealed with an anti‐fluorescent quencher. The images were taken under a fluorescence microscope (Olympus BX53 biological microscope).

### 2.21 Methylation‐specific quantitative PCR (qMSP)

qMSP was used to determine the methylation levels of NRF2 promoter using TaqMan‐based technology in a Lightcycler LC480 system (Roche Applied Science). Total DNAs underwent bisulfite conversion using the EZ DNA Methylation‐Lightning Automation Kit (Zymo Research) according to the manufacturer's instructions. CpG islands in the NRF2 and HMOX1 promoters were predicted, and primers for MSP were designed using MetPrimer online analyser. The designed primers are listed in the Supporting Information (Table S2). Amplification reactions were performed in triplicate in a total volume of 20 µL that contained 50 ng of bisulphite‐modified DNA, 600 nM forward and reverse primers, and 10 µL of QuantiTect 2 × SYBR Green PCR mix (Invitrogen, Inc.). PCR conditions were as follows: 95°C for 15 min, followed by 40 cycles of denaturing at 95°C for 30 s, annealing at 58°C for 30 s and elongation at 72°C for 30 s. Cycle threshold values were obtained for both the methylated‐specific (M) and unmethylated‐specific (U) primer sets, and percent methylation was calculated for each sample using the following formula: % methylation  =  100/(1 + 2^ΔCt(M‐U)^)%.^27^

### 2.22 In vivo animal experiment

GIST cell line‐based xenograft (CDX) and patient‐derived xenograft (PDX) models were performed in this study. 5‐6‐week‐old female BALB/c nude mice were purchased from the Vital River Laboratory Animal Technology Co., Ltd. All mice were housed in specific‐pathogen‐free conditions. A total number of 4 × 10^6^ GIST‐T1‐IR cells were injected subcutaneously into the left flank of mice. When the tumour was palpable, mice were randomly divided into indicated groups and treated with indicated drugs. Vehicle (negative control) and imatinib were administered orally (25 mg/ kg), and β‐elemene (50 mg/ kg) was administered intraperitoneally once every 3 days. In the due day, mice were sacrificed, and tumours were excised for analysis of their sizes and their weights.

The PDX models were initially generated using fresh tumour samples from patients with imatinib‐resistant GIST that were subcutaneously implanted into the dorsal flank of mice as the first generation (F0). Once an appropriate volume was reached, the tumours were excised, divided into equal pieces and subcutaneously implanted into mice as the second generation (F1). When the tumour was palpable, mice were randomly divided into indicated groups and treated with IM or β‐elemene or their combination as previously described. Tumour volumes were calculated every 3 days according to the formula volume = (length × width^2^) × 1/ 2 as described in the previous study. In the due day, mice were sacrificed, and tumours were excised for analysis of their sizes and their weights.

All procedures, following the National Institutes of Health Guide for the Care and Use of Laboratory Animals (NIH Publications No. 8023, revised 1978), were approved by the Sun Yat‐sen University Animal Care and Use Committee (L102012022120C, Guangzhou, China).

### 2.23 Statistical analysis

The data were analysed by GraphPad Prism 9 software in this study. All the results were represented as mean ± standard deviation. Comparisons were performed using unpaired Student's *t*‐test between the two groups. Multiple group comparisons were performed by using one‐way ANOVA. A *p*‐value of less than .05 is considered to be statistically significant.

## 3 RESULTS

### 3.1 Imatinib resistance is associated with ferroptosis activity in GIST

**FIGURE 1:**
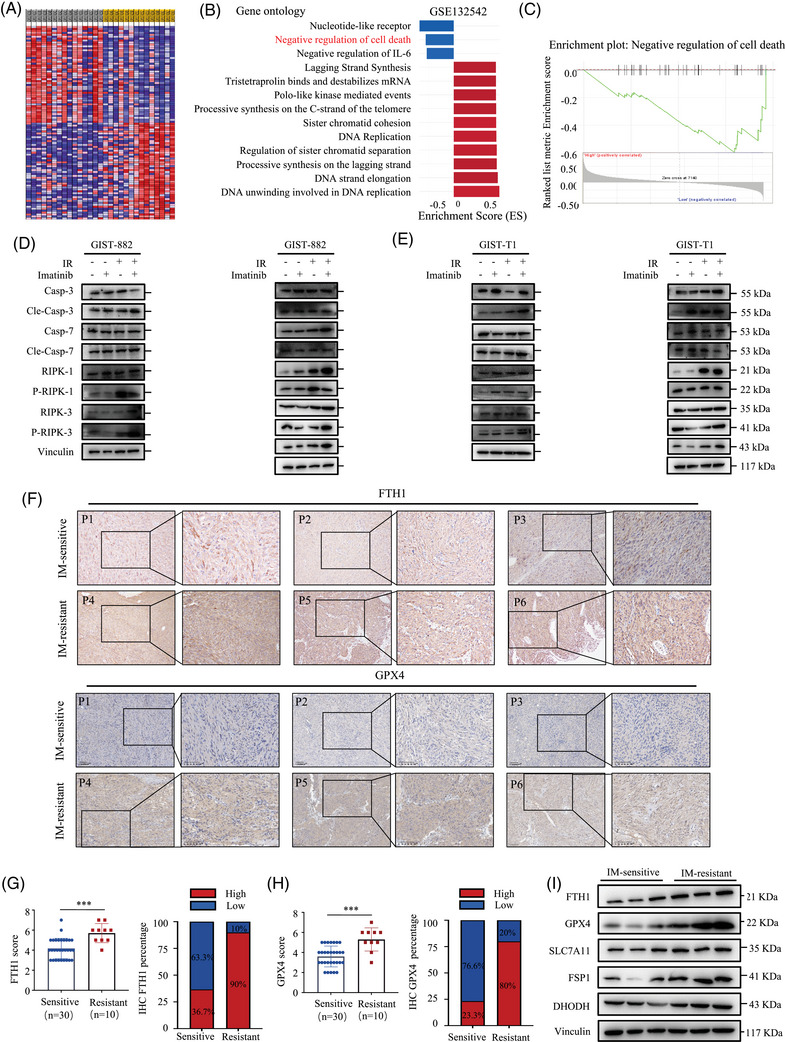
Imatinib resistance is associated with ferroptosis activity in gastrointestinal stromal tumour (GIST). (A) Heatmap of differentially expressed genes (DEGs) in imatinib‐naïve and ‐resistant GISTs from the GSE132542 dataset. (B) Gene ontology analysis of DEGs between imatinib‐naïve and ‐resistant GISTs from the GSE132542 dataset (all absolute log2 fold change > .5, false discovery rate (FDR) < 10%). (C) Gene set enrichment analysis (GSEA) plots of negative regulation of cell death signalling pathway. (D) Western blotting analysis of caspase‐3, cleaved caspase‐3, caspase7, cleaved caspase‐7, phosphorylated RIP1, total RIP1, phosphorylated RIP3, total RIP3, GSDME, cleaved GSDME, GSDMD, cleaved GSDMD, ferritin heavy chain (FTH1) and glutathione peroxidase 4 (GPX4), solute carrier family 7 member 11 (SLC7A11), FSP1 and dihydroorotate dehydrogenase (DHODH) expression in parental and imatinib‐resistant (IR) GIST‐882 cells treated with 882 20 µM imatinib for 24 h (for GIST‐882 and 20 nM for GIST‐T1, 24 h). Vinculin was included as a loading control. (E) Western blotting analysis of caspase‐3, cleaved caspase‐3, caspase7, cleaved caspase‐7, phosphorylated RIP1, total RIP1, phosphorylated RIP3, total RIP3, GSDME, cleaved GSDME, GSDMD, cleaved GSDMD, FTH1 and GPX4, SLC7A11, FSP1 and DHODH expression in parental and IR GIST‐T1 cells treated with 20 nM imatinib for 24 h. Vinculin was included as a loading control. (F) The expression of FTH1 and GPX4 in imatinib‐sensitive and imatinib‐resistant GIST specimens using immunohistochemistry (IHC) staining assay (Mann–Whitney U test). (G) Statistics of FTH1 expression in imatinib‐sensitive (*n* = 30) and imatinib‐resistant GIST specimens (*n *= 10) and percentage of GIST specimens with high or low FTH1 expression. (H) Statistics of GPX4 expression in imatinib‐sensitive (*n* = 30) and imatinib‐resistant GIST specimens (*n* = 10) and percentage of GIST specimens with high or low GPX4 expression. (I) Western blotting analysis showing the protein levels of FTH1, GPX4, SLC7A11, FSP1 and DHODH in six GIST specimens, including three samples from patients with imatinib‐treated PD(Progressive Disease) and three samples from patients with imatinib‐treated PR(Partial Response). Data represent the mean ± standard deviation (SD); *p < .05; **p < .01; ***p < .001. An unpaired *t*‐test was used unless otherwise stated.

To explore the mechanism of imatinib resistance in GIST, GSEA was performed to compare the gene microarray profiles in GIST samples. GSE 132542 dataset contains 29 samples, which are divided into two groups according to the sensitivity of imatinib: imatinib‐naïve (*n* = 14) and imatinib‐resistant (*n* = 15). A heatmap showed DEGs (Figure 1A). Gene ontology analysis of DEGs of imatinib‐resistant/imatinib‐naïve revealed a significant enrichment in negative regulation of cell death (Figure 1B,C).

To further investigate the relationship between the regulation of cell death and imatinib response in GISTs, imatinib‐resistant GIST‐882 and GIST‐T1 cells were established, and their imatinib‐resistant characteristics were confirmed by the IC50 of imatinib. As shown in Figure S1B,C (Supporting Information), the IC50 of imatinib was significantly higher in GIST‐882‐IR and GIST‐T1‐IR cells than the corresponding parental cells (36.08 ± 1.66 vs.15.65 ± 1.61 µM and 34.28 ± 12 vs.7.824 ± 2.35 nM, respectively).

To confirm the type of cell death programs that regulated imatinib resistance, we further investigated these common cell death pathways, including apoptosis, necroptosis, pyroptosis and ferroptosis. We evaluated the levels of relevant markers at the molecular level in the imatinib‐resistant GIST cells and corresponding parental cells during imatinib treatment. We found that well‐established ferroptosis markers were significantly upregulated, such as FTH1, GPX4, SLC7A11, S100 calcium binding protein A4 (FSP1) and DHODH (Figure 1D,E). For apoptosis, the results revealed no obvious effect on caspase‐3 and caspase‐7 in imatinib‐resistant cells, but activation of cleaved caspase‐3 and caspase‐7 was observed in parental cells with imatinib treatment. This result is consistent with the previous study. Accordingly, the markers of necroptosis exhibited similar patterns. We noted enhanced levels of phosphorylated RIPK1 and RIPK3 in GIST‐882‐IR but no obvious difference after imatinib treatment. There is no obvious difference between phosphorylated RIPK1 and RIPK3 in GIST‐T1 cells. Next, we explored the impact of imatinib treatments on markers of pyroptosis. GSDMD and GSDME are the main executioners that form membrane pores under specific conditions. The results revealed no obvious effect on the cleavage of GSDME and GSDMD (Figure 1D,E). Altogether, these data indicate that imatinib resistance in GIST cells may be associated with the negative regulation of ferroptosis activity.

We also investigate the expression of ferroptosis‐relevant markers in clinical samples. IHC staining revealed that the expression of FTH1 and GPX4 in imatinib‐resistant GIST clinical samples was higher than that in imatinib‐sensitive samples. Moreover, the proportion of FTH1 and GPX4 high expression in imatinib‐resistant GIST samples is greater than that in imatinib‐sensitive samples, confirmed by the quantification of IHC staining (Figure 1G,H). Additionally, western blotting validates the expression of FTH1, GPX4, SLC7A11, FSP1 and DHODH in fresh surgical tumour tissues from three imatinib‐resistant patients compared with three imatinib‐sensitive patients receiving neoadjuvant imatinib therapy (Figure 1I). Altogether, these data indicate that imatinib resistance in GIST cells may be associated with negative regulation of ferroptosis activity.

### 3.2 Ferroptosis activity is suppressed in acquired imatinib‐resistant GIST cells and inducing ferroptosis can promote imatinib sensitivity

**FIGURE 2:**
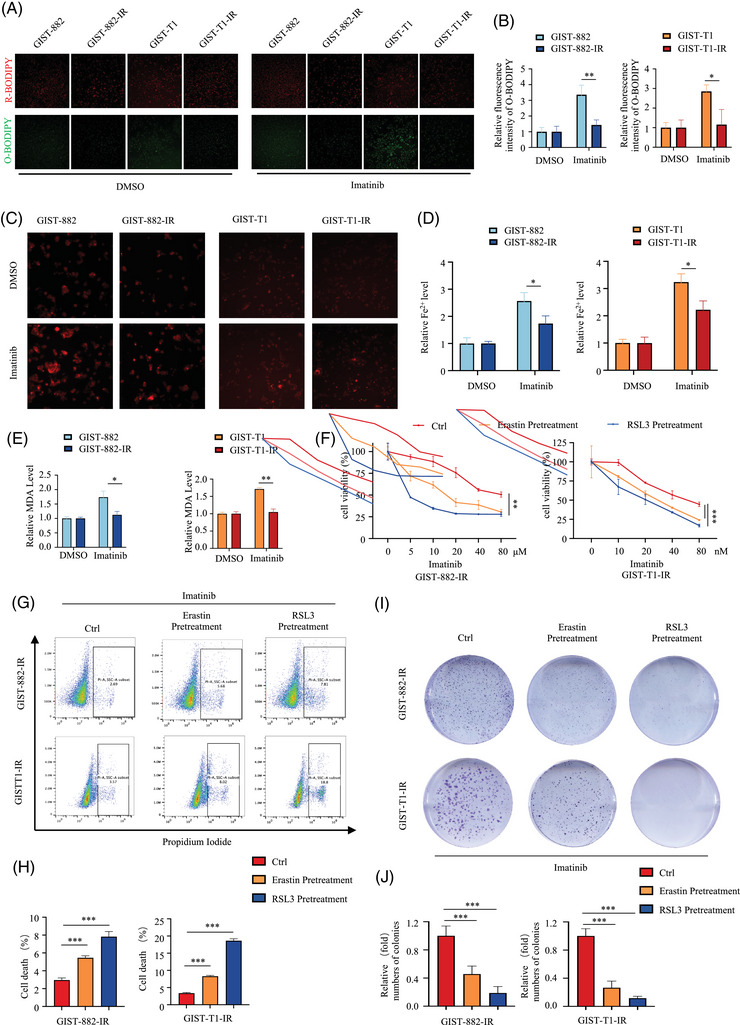
Ferroptosis activity is suppressed in acquired imatinib‐resistant GIST cells and inducing ferroptosis can promote imatinib sensitivity. (A) Representative fluorescent images of lipid peroxidation detected by BODIPY (581/591) C11 probe in GIST‐882, GIST‐882‐IR, GIST‐T1 and GIST‐T1‐IR with or without treatment of imatinib for 24 h. Scale bar = 100 µm. (B) Quantification of relative fluorescence intensity of oxidised BODIPY by Image J. (C) The Fe^2+^ levels detected by FerroOrange fluorescence probe in GIST‐882, GIST‐882‐IR, GIST‐T1 and GIST‐T1‐IR cells with or without treatment of imatinib. (D) Quantification of relative fluorescence intensity of Fe^2+^ levels by Image J. (E) The malondialdehyde (MDA) levels detected in GIST‐882, GIST‐882‐IR, GIST‐T1 and GIST‐T1‐IR cells with or without treatment of imatinib. (F) Cell viability of imatinib‐exposed GIST‐882‐IR and GIST‐T1‐IR pretreated with erastin and RAS‐selective lethal 3 (RSL3) or not was detected via CCK‐8 assay. (G) Cell death measurements were taken using flow cytometry analysis and statistical histograms of propidium iodide (PI)‐positive cells in IM‐exposed GIST‐882‐IR and GIST‐T1‐IR, pretreated with erastin and RSL3. (H) Statistical analysis of the cell death. All experiments were performed in triplicate, and relative colony numbers are shown as means ± SD. (I) Antitumour effects of imatinib in imatinib‐exposed GIST‐882‐IR and GIST‐T1‐IR pretreated with erastin and RSL3 or not were evaluated by colony formation assay. (J) Statistical analysis of the colony formation assay. All experiments were performed in triplicate. Data represent the mean ± SD; *p < .05; **p < .01; ***p < .001. An unpaired *t*‐test was used unless otherwise stated.

Previous researches have also highlighted the critical role of ferroptosis in the development of resistance to IM in GIST,^28^, ^29^, ^30^ and imatinib induces ferroptosis in GIST by promoting STUB1‐mediated GPX4 ubiquitination.^31^ Here, we evaluated the sensitivity of GIST‐882‐IR and GIST‐T1‐IR to ferroptosis relative to their parental cells at the same concentration of imatinib treatment. We found that the intracellular lipid peroxides, Fe^2+^ and MDA of GIST‐882‐IR and GIST‐T1‐IR cells were significantly lower than those of the corresponding parental cell lines (Figure 2A–E). These results suggested that the ferroptosis activity is inhabited in GIST‐882‐IR and GIST‐T1‐IR cells.

A growing number of evidence indicates that the induction of ferroptosis can inhibit tumour growth and overcome drug resistance.^32^ Here, we tested whether the pre‐treatment with ferroptosis inducer erastin and RSL3 can enhance the sensitivity of GIST‐882‐IR and GIST‐T1‐IR cells to imatinib. Before treating cells with imatinib, we pretreated cells with erastin (1 µM) and RSL3 (.5 µM) for 6 h. This pretreatment produced no increase in cell death in GIST cells (Figure S2A,B). As shown in Figure 2F, pretreatment of erastin and RSL3 promoted the cytotoxic and inhibitory effects of imatinib on GIST‐882‐IR and GIST‐T1‐IR cells. Consistently, colony formation (Figure 2G,H) and cell death assay (Figure 2I,J) showed that pretreatment of erastin and RSL3 significantly increased the sensitivity of imatinib in GIST‐882‐IR and GIST‐T1‐IR cells. Taken together, the above findings indicated that inducing ferroptosis can promote imatinib sensitivity in imatinib‐resistant GIST cells. This led us to speculate that ferroptosis could be a strategy for susceptibility to imatinib‐based chemotherapy.

### 3.3 β‐elemene induces ferroptosis in imatinib‐resistant GIST cells

**FIGURE 3:**
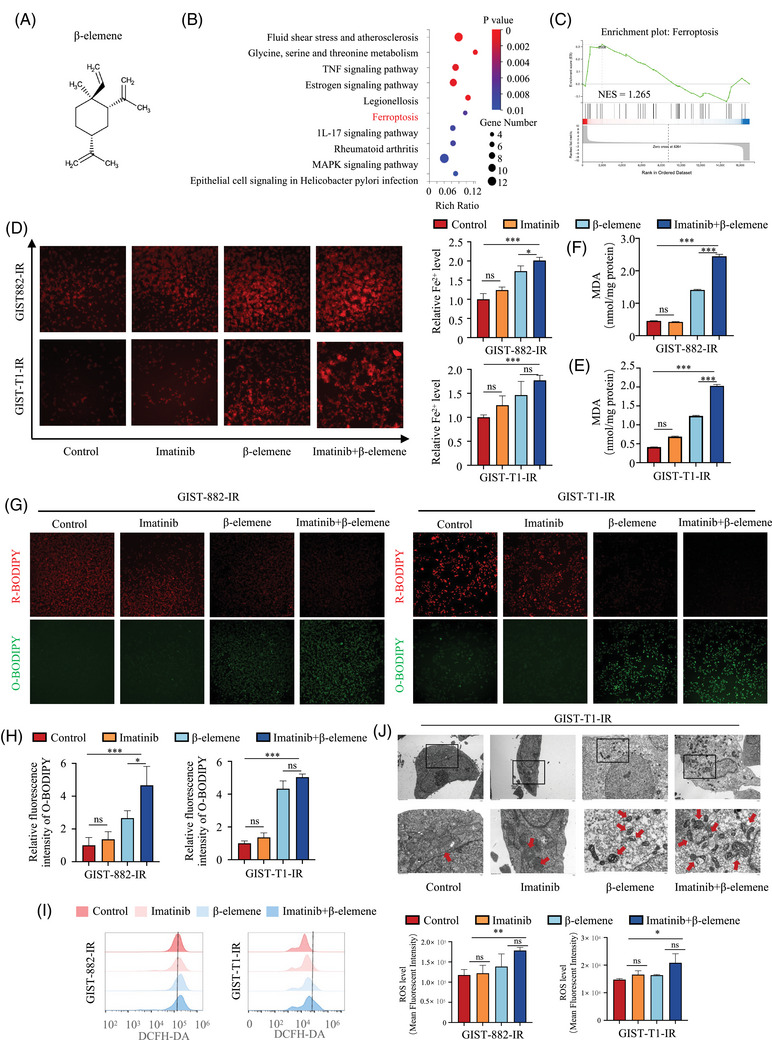
β‐elemene induces ferroptosis in imatinib‐resistant GIST cells. (A) Chemical structure of β‐elemene. (B‐C) Kyoto Encyclopedia of Genes and Genomes pathway enrichment analysis and GSEA showed that genes involved in ferroptosis were significantly dysregulated under β‐elemene treatment. (D) The Fe^2+^ levels detected by FerroOrange fluorescence probe in GIST‐882‐IR and GIST‐T1‐IR cells treated with DMSO, imatinib, β‐elemene or imatinib+β‐elemene for 24 h. (E) Quantification of relative fluorescence intensity of Fe^2+^ levels by Image J. (F) The MDA levels detected in GIST‐882‐IR and GIST‐T1‐IR cells treated with DMSO, imatinib, β‐elemene or imatinib+β‐elemene for 24 h. (G) Representative fluorescent images of lipid peroxidation detected by BODIPY (581/591) C11 probe in GIST‐882‐IR and GIST‐T1‐IR cells treated with DMSO, imatinib, β‐elemene or imatinib+β‐elemene for 24 h. Scale bar = 100 µm. (H) Quantification of relative fluorescence intensity of oxidised BODIPY by Image J. (I) The ROS accumulation was detected using flow cytometry analysis and statistical histograms of positive cells in GIST‐882‐IR and GIST‐T1‐IR cells treated with DMSO, imatinib, β‐elemene or imatinib+β‐elemene for 24 h. (J) Representative cell ultrastructural images of GIST‐T1‐IR cells treated with DMSO, imatinib, β‐elemene or imatinib+β‐elemene for 24 h. All experiments were performed in triplicate. Data represent the mean ± SD; *p < .05; **p < .01; ***p < .001. An unpaired *t*‐test was used unless otherwise stated.

A previous study demonstrated that β‐elemene can induce ferroptosis.^22^ We hypothesise that β‐elemene may also enhance the sensitivity of imatinib by inducing ferroptosis. To further investigate whether the combination of β‐elemene and imatinib promotes the imatinib sensitivity in GIST‐882‐IR and GIST‐T1‐IR cells by inducing ferroptosis, we conducted high‐throughput RNA‐seq to compare the gene expression profile of imatinib and/or β‐elemene‐treated GIST‐T1‐IR cells. Compared with cells treated with imatinib alone, the co‐treatment of β‐elemene and imatinib induced changes in the intracellular expression profile. KEGG pathway enrichment analysis and GSEA revealed that the DEGs after dual‐drug treatment were enriched in the ferroptosis pathway (Figure 3B,C). The results suggested that β‐elemene could indeed cause ferroptosis in GIST cells.

Important aspects of ferroptosis‐mediated cell death are characterised by ROS, Fe^2+^ and lipid peroxides and MDA accumulation.^33^ To verify this possibility, we assessed ROS, Fe^2+^ and lipid peroxides and MDA levels in imatinib‐resistant cells. By immunofluorescence examination, a promoted FerroOrange fluorescence was recorded in the β‐elemene plus imatinib‐treated GIST cells, indicating that co‐treatment with β‐elemene and imatinib promoted the accumulation of Fe^2+^ in GIST‐882‐IR and GIST‐T1‐IR cells (Figure 3D,E). The lipid peroxidation level was determined by fluorescent probe BODIPY 581/591 C11. As expected, treatment with β‐elemene or imatinib alone had only a slight effect, but combined treatment with β‐elemene and imatinib significantly increased both the intracellular lipid peroxidation level and MDA level (Figure 3F–H). The lipid ROS, a specific index of ferroptosis, was also examined, and our results showed a significant increase in ROS levels in GIST‐882‐IR and GIST‐T1‐IR cells after β‐elemene plus imatinib treatment (Figure 3I). Moreover, we used a TEM to observe changes in the mitochondrial ultrastructure of the cells. As shown in Figure 3J, red arrows indicated that GIST‐T1‐IR co‐treated with imatinib and β‐elemene shrunken mitochondria with increased membrane densities, or disappeared mitochondrial cristae, which were characteristic morphological features of ferroptosis. In conclusion, these results strongly suggest that the co‐treatment of β‐elemene and imatinib can induce ferroptosis in imatinib‐resistant GIST cells.

### 3.4 β‐elemene promotes imatinib sensitivity in imatinib‐resistant GISTs through ferroptosis

**FIGURE 4:**
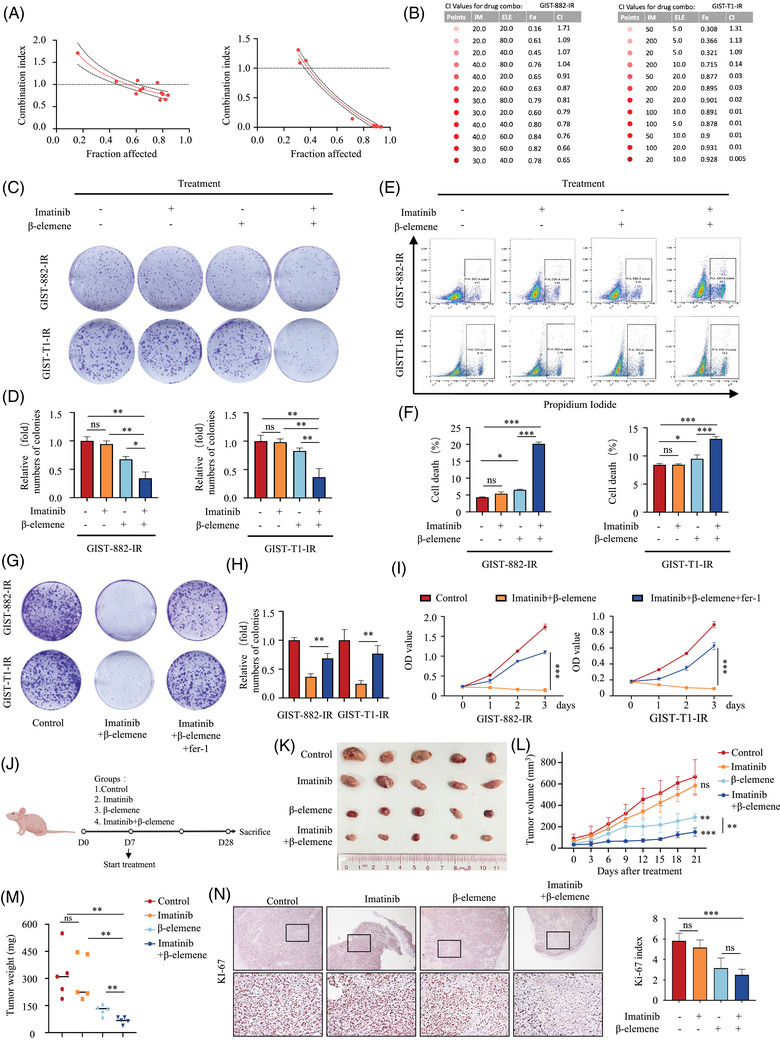
β‐elemene promotes imatinib sensitivity in imatinib‐resistant GISTs through ferroptosis. (A) The interaction between imatinib and β‐elemene on cell cytotoxicity was examined by the median‐effect method of Chou–Talalay. (B) The dose and combination index of imatinib in combination with β‐elemene on GIST‐882‐IR and GIST‐T1‐IR cells was estimated by calculation of combination index (CI) values using Compusyn software. The darker point indicates a stronger synergistic effect. The fraction affected values indicate the percentage of cell inhibition, while the CI values indicate the effects of combination treatments. (C) Antitumour effects of imatinib and β‐elemene in GIST‐882‐IR and GIST‐T1‐IR cells after the indicated treatment were evaluated by colony formation assay. (D) Statistical analysis of the colony formation assay. (E) Representative results of PI‐positive cells in IM‐exposed GIST‐882‐IR and GIST‐T1‐IR after the indicated treatment and quantitative analysis after the treatment for 48 h. (F) Statistical histogram of the flow cytometry cell death analysis. (G) Antitumour effects of GIST‐882‐IR and GIST‐T1‐IR cells treated with or without ferroptosis inhibitor Ferrostatin‐1 (Fer‐1) were evaluated by colony formation assay. (H) Statistical analysis of the colony formation assay of GIST‐882‐IR and GIST‐T1‐IR cells treated with or without ferroptosis inhibitor Fer‐1. (I) Cell viability of GIST‐882‐IR and GIST‐T1‐IR cells treated with or without ferroptosis inhibitor Fer‐1. (J) Schematic description of the in vivo anticancer effect of combined treatment with β‐elemene and imatinib in the cell line‐based xenograft model. (K) Photograph and comparison of tumour sizes in the indicated groups. (L) Growth curve of GIST‐T1‐IR xenografts in the indicated groups. (M) The tumour weight of GIST‐T1‐IR xenografts in the indicated groups. (N) Representative images of IHC staining of MKI67/K‐67 in mouse tumours. All experiments were performed in triplicate. Data represent the mean ± SD; *p < .05; **p < .01; ***p < .001. An unpaired *t*‐test was used unless otherwise stated.

To determine the synergistic antitumour effects of β‐elemene and imatinib in GIST, we evaluated the viability of GIST‐882‐IR and GIST‐T1‐IR cells by treating them with different concentrations of drug combinations for 48 h. Cell cytotoxicity was measured by CCK8 assay, and the CI was calculated according to the Chou–Talalay method (Figure 4A). We calculated CI values for each combination treatment to assess whether the combination of β‐elemene and imatinib showed synergistic enhancement of cell cytotoxicity. The CI values for β‐elemene and imatinib for GIST‐882‐IR (imatinib, 30 µM; β‐elemene, 40 µg/L) and GIST‐T1‐IR (imatinib, 20 nM; β‐elemene, 10 µg/L) were .65 and .005, respectively, at 48 h and were the greatest synergistic effects observed for the assessed drug concentrations (Figure 4B). In addition, the combination of the two drugs induced a significant reduction in colony formation, compared with the use of each drug alone (Figure 4C,D). In order to obtain objective quantification of cell death, we carried out a PI staining assay followed by flow cytometry. The PI staining assay showed that a significant increase in the number of dead cells was observed in GIST‐882‐IR and GIST‐T1‐IR cells when exposed to β‐elemene in combination with imatinib (Figure 4E,F). Further, the ferroptosis inhibitor, Fer‐1 markedly blocked the effects of the combination treatment on colony formation (Figure 4G,H) and cell viability (Figure 4I). These results show that β‐elemene and imatinib exhibit synergistic lethal effects through ferroptosis.

To assess whether our in vitro results could be reproduced in vivo, we generated a GIST CDX model from GIST‐T1‐IR cells (Figure 4J). Consistent with our results in vitro, imatinib and β‐elemene exhibited synergistic effects in the CDX model. Results show that both imatinib and β‐elemene alone were found to slightly reduce tumour growth, while combined treatment with imatinib and β‐elemene completely suppressed tumour growth in vivo (Figure 4K–M). Combined treatment with imatinib and β‐elemene significantly reduced the growth rate of GIST tumours as shown by IHC staining (Figure 4N). Taken together, these results show that imatinib and β‐elemene exhibit synergistic lethal effects through ferroptosis.

### 3.5 β‐elemene induced ferroptosis in imatinib‐resistant GIST cells through HMOX1

**FIGURE 5:**
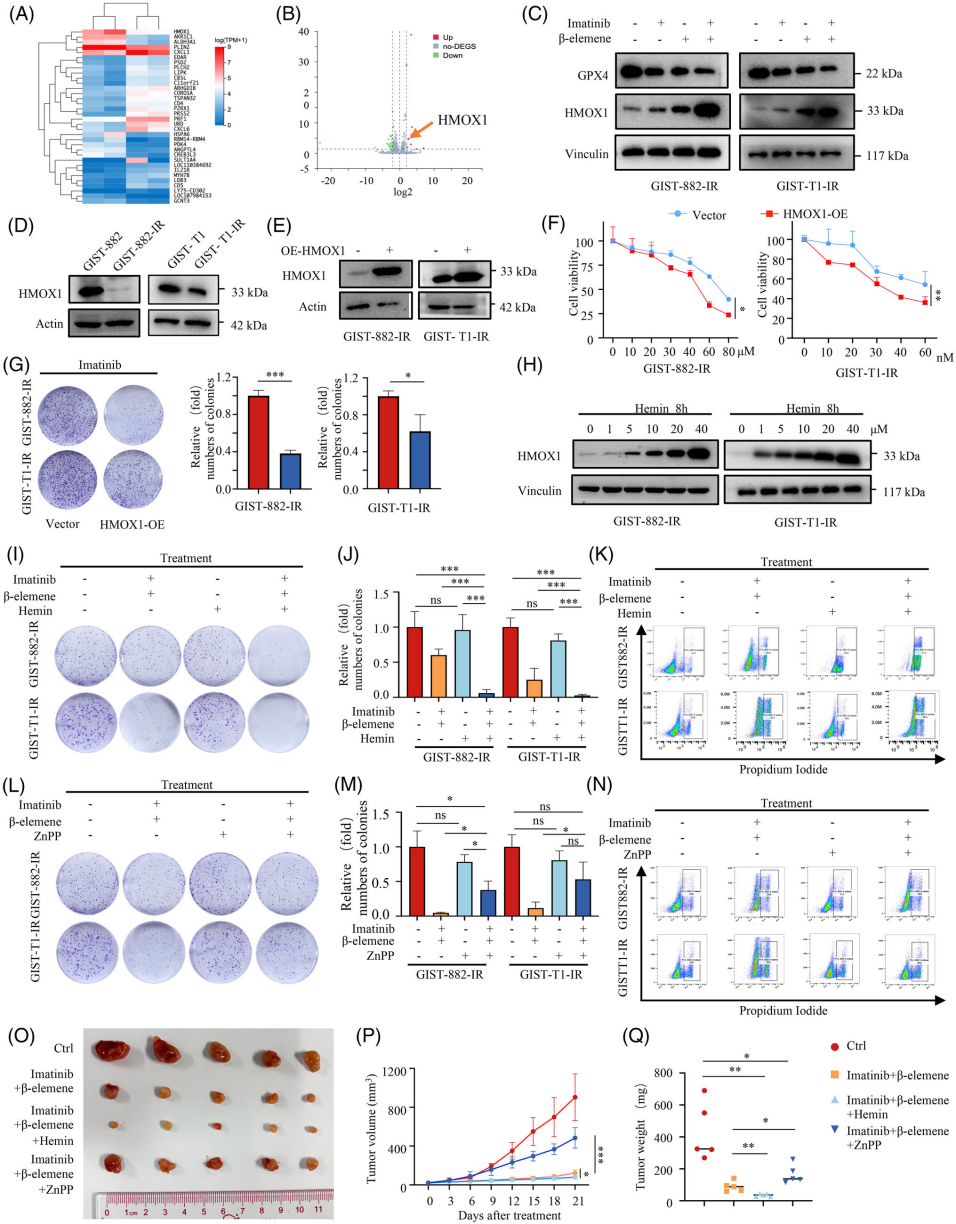
β‐elemene induced ferroptosis in imatinib‐resistant GISTs through HMOX1. (A) Heatmap of the RNA‐seq analysis results for GIST‐T1‐IR cells treated with DMSO or β‐elemene. (B) Volcano plot of down‐regulated and upregulated for GIST‐T1‐IR cells treated with DMSO or β‐elemene. (C) Western blotting analysis of HMOX1 and GPX4 expression in GIST cells treated with imatinib, β‐elemene or imatinib+β‐elemene. (D) Western blotting analysis of HMOX1 expression in parental and imatinib‐resistant GIST cells. (E) Western blotting analysis of HMOX1 in GIST‐882‐IR and GIST‐T1‐IR cells transfected with HMOX1‐expressing plasmid. (F) CCK‐8 assay of sensitivity to imatinib in HMOX1‐overexpressed GIST‐882‐IR and GIST‐T1‐IR cells versus control GIST‐882_IR and GIST‐T1‐IR cells. (G) The colony formation assay of sensitivity to imatinib in HMOX1‐overexpressed GIST‐882‐IR and GIST‐T1‐IR cells versus control GIST‐882_IR and GIST‐T1‐IR cells. (H) Western blot showing changes in HMOX1 expression in response to Hemin. (I‐J) The colony formation assay and statistical histogram of GIST‐882‐IR and GIST‐T1‐IR cells treated with imatinib+β‐elemene with or without hemin. (K) Cell death measurements by flow cytometry analysis in GIST‐882‐IR and GIST‐T1‐IR cells treated with imatinib+β‐elemene with or without hemin. (L–M) The colony formation assay and statistical histogram of GIST‐882‐IR and GIST‐T1‐IR cells treated with imatinib+β‐elemene with or without zinc protoporphyrin‐9 (ZnPP). (N) Cell death measurements by flow cytometry analysis in GIST‐882‐IR and GIST‐T1‐IR cells treated with imatinib+β‐elemene with or without ZnPP. (O) Photograph and comparison of tumour sizes in different groups. (P) Growth curve of GIST‐T1‐IR xenografts in the indicated groups. (Q) The tumour weight of GIST‐T1‐IR xenografts in the indicated groups. All experiments were performed in triplicate. Data represent the mean ± SD; *p < .05; **p < .01; ***p < .001. An unpaired *t*‐test was used unless otherwise stated.

In order to further investigate the relationship between the differential gene expression profile and ferroptosis in GIST, we analysed our RNA‐seq data and found that HMOX1 expression was significantly increased in dual‐drug‐treated cells (Figure 5A,B). Western blot analysis showed that, compared with the control group and the single drug groups, the expression of HMOX1 in the dual drug groups increased significantly, while the expression of GPX4 decreased (Figure 5C). Moreover, HMOX1 expression significantly decreased in the imatinib‐resistant GIST cells, compared to their parental cells (Figure 5D).

Overexpression of HMOX1 has been shown to exert pro‐oxidant effects and to induce ferroptosis.^34^, ^35^ HMOX1 detoxifies heme into biliverdin, releasing carbon monoxide and Fe^2+^.^36^ We first established stable HMOX1 overexpressing cells from the GIST‐882‐IR and GIST‐T1‐IR cells (Figure 5E). The CCK‐8 and colon formation assay showed that HMOX1 overexpression significantly inhibited the proliferation of GIST‐882‐IR and GIST‐T1‐IR cells exposed to imatinib, and imatinib sensitivity could be reversed (Figure 5F,G). Addition of hemin induced ferroptosis more rapidly when treated in combination with imatinib and β‐elemene (Figure 5H–K). However, treatment with 10 µmol/L ZnPP, a specific inhibitor of HMOX1, attenuated cell death induced by the combination treatment (Figure 5L–N). Based on this finding, we tried to determine the therapeutic effect of the combination of these three drugs in vivo. We found that the antitumour activity of mice treated with imatinib, β‐elemene and 20 µM hemin was significantly increased, compared with those treated with the control group and with imatinib and β‐elemene groups. And ZnPP can attenuate the antitumour effects of the combination with imatinib and β‐elemene (Figure 5O–Q). These results further emphasise that HMOX1 plays an essential role in the ferroptosis in GIST cells, which is induced by β‐elemene.

### 3.6 β‐elemene targets N6AMT1 to promote imatinib sensitivity in imatinib‐resistant GIST cells via the NRF2/HMOX1 axis

**FIGURE 6:**
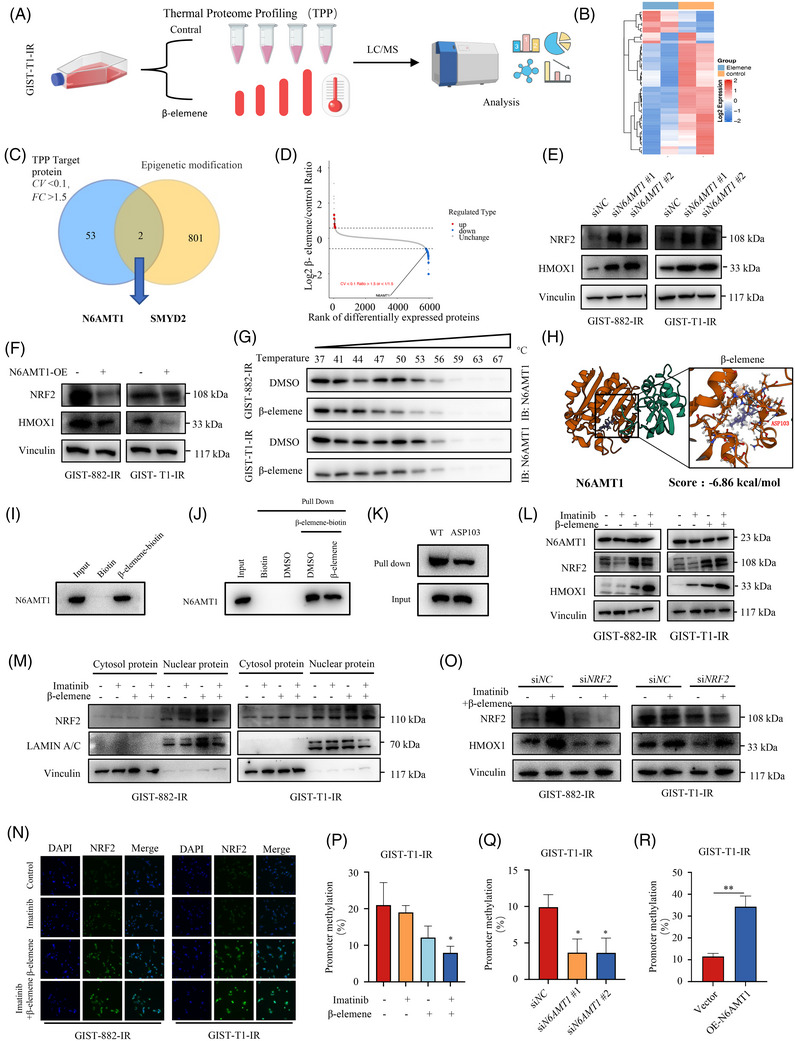
β‐elemene targets N6AMT1 to promote imatinib sensitivity in imatinib‐resistant GIST cells via the nuclear factor erythroid 2‐related factor 2 (NRF2)/HMOX1 axis. (A) The workflow for cellular targets identification of β‐elemene in GIST‐T1‐IR by thermal proteome profiling (TPP). (B) Heatmap of differentially expressed protein in GIST‐T1‐IR cells between control and β‐elemene‐treated groups. (C) Venn diagram of β‐elemene target screening. (D) Volcano plot of cellular targets of β‐elemene by the TPP strategy. (E) Western blot analysis of NRF2 and HMOX1 in GIST cells with or without N6AMT1 knockdown by small interfering RNA (siRNA). (F) Western blot analysis of NRF2 and HMOX1 in GIST cells with or without N6AMT1 overexpression. (G) Cellular thermal shift assay demonstrated a stabilisation effect of N6AMT1 with β‐elemene. (H) The interaction between β‐elemene with N6AMT1 targeting Asp103 was predicted by molecular docking. (I) GIST‐T1‐IR cell lysates were incubated with either biotin or β‐elemene‐biotin at 4°C overnight, and then a pulldown assay was performed. (J) GIST‐T1‐IR cell lysates were preincubated with either DMSO or free β‐elemene, followed by subsequent incubation with β‐elemene‐biotin. The interaction between N6AMT1 and β‐elemene was then detected by capturing β‐elemene‐biotin. (K) The mutant N6AMT1 proteins were incubated with β‐elemene, followed by protein affinity pull‐down assay and detected by immunoblotting. (L) Western blot analysis of N6AMT1/NRF2/HMOX1 axis in GIST cells treated with imatinib, β‐elemene or imatinib+β‐elemene. (M) Western blot analysis of NRF2 in cytosol protein and nuclear protein. (N) The staining intensity and localisation of NRF2 in the indicated groups were analysed by immunofluorescence staining. (O) Western blot analysis of the relationship of NRF2 and HMOX1 in ferroptosis with knockdown of NRF2 in GIST cells followed by imatinib+β‐elemene treatment. (P) Methylation level of NRF2 promoter in GIST‐T1‐IR cell treated with imatinib, β‐elemene or imatinib+β‐elemene. (Q) Methylation level of NRF2 promoter in GIST‐T1‐IR cell with or without N6AMT1 knockdown by siRNA. (R) Methylation level of NRF2 promoter in GIST‐T1‐IR cell with or without N6AMT1 overexpression. Data represent the mean ± SD; *p < .05; **p < .01; ***p < .001. An unpaired *t*‐test was used unless otherwise stated.

A high expression of HMOX1 in GIST cells treated with β‐elemene and imatinib is a key factor in ferroptosis activation. But the specific mechanism of HMOX1 high expression under β‐elemene treatment is still unclear. Here, we conducted the TPP assay to identify the potential targets of β‐elemene and explore the mechanisms of regulating HMOX1 expression (Figure 6A). TPP is an emerging technique that identifies ligand‐induced shifts of thermal stability in a high‐throughput manner, making it a great tool for tracking drug targets in living cells. As shown in Figure 6B and Table S3, we overall identified 6063 proteins, out of which 53 exhibited high confidence (FC > 1.5; *p*‐value < .05, CV < .1). KEGG pathway analysis showed that β‐elemene was associated with cell growth and death pathway and drug resistance: antineoplastic pathway, which is consistent to our research (Figure S3A). Functional enrichment analysis revealed that β‐elemene was closely associated with methyltransferase activity, giving us clues to further investigate (Figure S3B).

Epigenetic modifications have been reported to play a critical role in the regulation of ferroptosis.^37^, ^38^ Moreover, β‐elemene can reverse gefitinib resistance in non‐small cell lung cancer through m6A methylation modification‐mediated autophagy.^20^ Based on these previous studies, we speculate that β‐elemene may regulate the process of ferroptosis through epigenetic regulation. Among these 53 potential target proteins, two epigenetic modifications related protein N6AMT1 and SMYD2 have been identified (Figure 6C,D). In particular, there was no correlation between SMYD2 knockout and HMOX1 expression (Figure S4A,B). The expression of HMOX1 is related to knockdown or overexpression of N6AMT1 (Figure 6E,F). Therefore, we think that N6AMT1 may be a potential target of β‐elemene.

We validated the interaction of β‐elemene with N6AMT1 in GIST‐882‐IR and GIST‐T1‐IR cells by the CETSA, and our result showed that β‐elemene significantly decreased the stability of N6AMT1 by promoting a thermal shift (Figure 6G). Molecular docking was performed to provide deeper insight into the interactions between β‐elemene and N6AMT1. Results showed that β‐elemene bound to N6AMT1 protein targets through visible hydrogen bonds and strong electrostatic interactions. To identify the binding site of β‐elemene, docking simulations were performed to model its interaction with each amino acid residue. As shown in Figures 6H and S5A,B, β‐elemene covalently bound to the N6AMT1 protein by contacts with scores of −6.68 kcal/mol for Asp103, −5.309 kcal/mol for Lys2 and −5.326 kcal/mol for Asn77.

We synthesised β‐elemene‐biotin to determine the target of β‐elemene in GIST cells. The GIST‐T1‐IR cell lysates were subsequently treated with β‐elemene‐biotin and biotin. Samples were analysed using an anti‐N6AMT1 antibody, verifying that β‐elemene precipitated N6AMT1. Furthermore, preincubation with free β‐elemene inhibited the interaction between β‐elemene‐biotin and N6AMT1 (Figure 6I,J). We further examined the interactions between N6AMT1 and β‐elemene in this binding state by mutagenesis studies, followed by pull‐down assays. As illustrated in Figures 6K and S5C,D, mutations at Lys2 and Asn77 exerted a lesser impact, compared to mutations at Asp103. In conclusion, these findings indicated that β‐elemene covalently associates with N6AMT1, specifically targeting Asp103 of the protein.

To evaluate whether N6AMT1 can affect the ferroptosis effect of β‐elemene, we initially knocked down N6AMT1 expression using a siRNA and N6AMT1 overexpression in GIST‐882‐IR and GIST‐T1‐IR cells (Figure S6A,E). Knockdown of N6AMT1 resulted in a substantial reduction of cell viability in GIST‐882‐IR and GIST‐T1‐IR cells exposed to imatinib (Figure S6B). Cell death assay revealed that N6AMT1 knockdown led to an increased level of PI‐positive cells (Figure S6C). Moreover, the Fe^2+^ levels were recorded to be increased after N6AMT1 knockdown (Figure S6D). However, when N6AMT1 was overexpressed in these cells, the inhibitory effect of β‐elemene combined with imatinib on cell viability and cell death assay was less pronounced (Figure S6F,G). The Fe^2+^ levels were recorded to be increased after N6AMT1 overexpression (Figure S6H). Collectively, these results suggest that N6AMT1 overexpression can reverse the inhibitory effects of β‐elemene combined with imatinib, and conversely N6AMT1 knockdown enhances the inhibitory effects of imatinib, indicating that N6AMT1 is the target of β‐elemene contributing to the ferroptosis effect.

HMOX1 expression is mainly controlled by NRF2, a key transcription factor that promotes the transcription of HMOX1 to induce ferroptosis.^39^, ^40^ Thus, we monitored N6AMT1, NRF2 and HMOX1 levels in GIST cells exposed to the indicated drugs. As shown in Figure 6L, western blot analysis showed that the NRF2 levels were highly increased in the GIST cells with β‐elemene treatment, compared to control and imatinib alone, accompanied by the upregulation of HMOX1. Treatment with β‐elemene can increase NRF2 level in the nucleus, thus promoting HMOX1 expression (Figure 6M). Meanwhile, immunofluorescence staining also showed an increase in overall fluorescence intensity and fluorescence intensity in the nucleus in the GIST cells with β‐elemene treatment, compared to control and imatinib alone (Figure 6N). These results indicated that the expression of HOMX1 was associated with NRF2.

To demonstrate that the expression of HMOX1 is regulated by NRF2, we further knocked down NRF2 expression in GIST cells. Transfection with NRF2 siRNA inhibited the expression of HMOX1 (Figure 6O) and reversed the ferroptosis induced by treatment with β‐elemene and imatinib (Figure S7A,B). Therefore, it is feasible to show that the NRF2/HMOX1 pathway plays a critical role in β‐elemene‐mediated ferroptosis of GIST cells.

Next, we further investigate the mechanism involved in the regulation of HMOX1 by N6AMT1. We have found that β‐elemene can increase the expression of NRF2 and HOMX1, but N6AMT1 expression did not change in GIST‐882‐IR and GIST‐T1‐IR cells after β‐elemene treatment (Figure 6L) before. Regarding the fact that N6AMT1 has been reported as a methyltransferase,^41^ it is conceivable that N6AMT1 participates in ferroptosis through DNA hypermethylation of a certain transcription factor. Since β‐elemene treatment did not affect the expression level of N6AMT1, we speculate that since β‐elemene binds to N6AMT1, it may inhibit the binding of N6AMT1 to NRF2 promoter regions, thereby suppressing its transcriptional repression function. Thus, we assumed that β‐elemene was involved in NRF2 promoter methylation by inhibiting N6AMT1 function. We then measured methylation levels of the NRF2 promoter in GIST cells treated with β‐elemene plus imatinib or alone. As anticipated, β‐elemene significantly reduced methylation of the NRF2 promoter and increased NRF2 gene expression in GIST cells (Figures 6P and S8A). In addition, inhibition of N6AMT1 by siRNA promoted the expression of NRF2 protein levels (Figure 6E) and reduced the methylation level of the NRF2 promoter (Figures 6Q and S8B). Conversely, overexpression of N6AMT1 triggered methylation of the NRF2 promoter (Figures 6R and S8C) and inhibited NRF2 gene expression in GIST cells (Figure 6F). In addition, we found that β‐elemene treatment, interference and overexpression of N6AMT1 had no obvious change on HMOX1 promoter (Figure S8D–I). These results indicate that β‐elemene target N6AMT1‐mediated DNA methylation of NRF2 promoter is a crucial factor in the regulatory axis of NRF2 and HMOX1. Collectively, these results suggest that β‐elemene may bind to N6AMT1 to disturb its activity and interaction with the effectors.

### 3.7 β‐elemene improves imatinib therapeutic efficiency in imatinib‐resistant GIST

**FIGURE 7:**
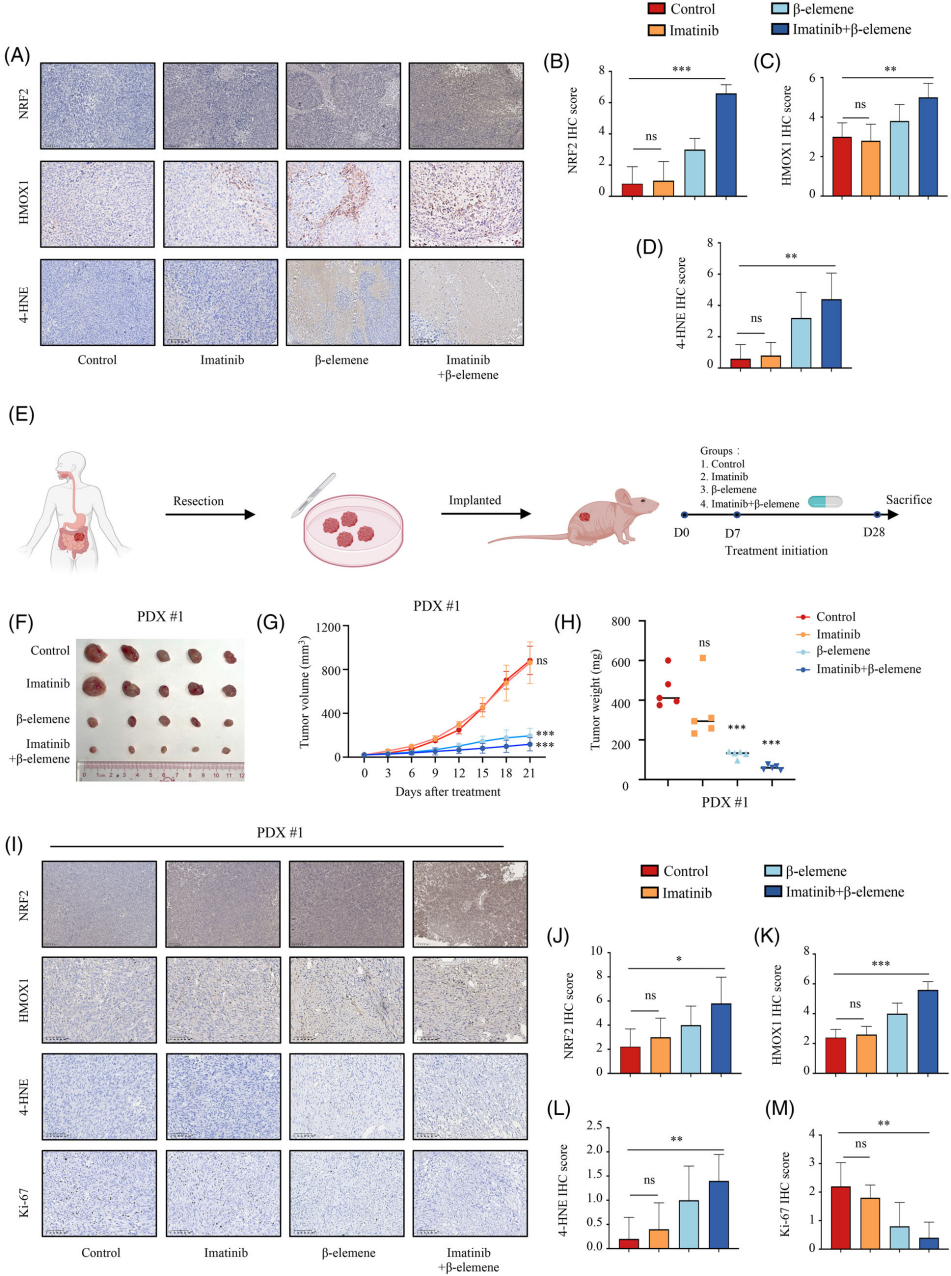
β‐elemene improves imatinib therapeutic efficiency in imatinib‐resistant GIST. (A–D) IHC staining of Nrf2, HMOX1 and 4‐HNE in tumour tissues generated by GIST‐T1‐IR cells‐based xenograft in the indicated groups. Scale bars, 50 µm. (E) Schematic description of the in vivo anticancer effect of combined treatment with imatinib and β‐elemene in the patient‐derived xenograft (PDX) model. (F) Photograph and comparison of tumour sizes of the PDX model in the indicated groups. (G) Growth curve of the PDX model in the indicated groups. (H) The tumour weight of the PDX model in the indicated groups. (I–M) IHC staining of 4‐HNE and HMOX1 in tumour tissues generated by the PDX model in the indicated groups. Scale bars, 50 µm. Data represent the mean ± SD; *p < .05; **p < .01; ***p < .001. An unpaired *t*‐test was used unless otherwise stated.

Last, preclinical evaluation of the effects of β‐elemene and imatinib treatment on imatinib resistance was carried out using animal models. We had evaluated the effect of β‐elemene and imatinib therapy in the CDX models before. Since β‐elemene induced ferroptosis via the NRF2/HMOX1 axis, we investigated the relevant marker in vivo model samples. Consistent with our proposed mechanism of study, β‐elemene and imatinib co‐treated tumours showed concomitant increases in NRF2, HMOX1 and 4‐HNE staining (Figure 7A–D).

Further, we established PDX models with better clinical relevance by using clinically imatinib‐resistant GIST tissues, followed by treatment with indicated drugs (Figure 7E). Similar to our CDX model, there was no statistical difference in both tumour size and weight between the control and imatinib groups, whereas the tumour was dramatically downsized in the β‐elemene and imatinib combination group (Figures 7F–H and S9A,B). Compared with the control or imatinib alone, imatinib combined with β‐elemene treatment increased HMOX1, NRF2 and 4‐HNE levels, along with a decrease in Ki‐67 expression (Figure 7J–M).

Collectively, these results suggest that β‐elemene synergistically enhances the antitumour effect of imatinib in GIST with imatinib resistance.

## 4 DISCUSSION

**FIGURE 8:**
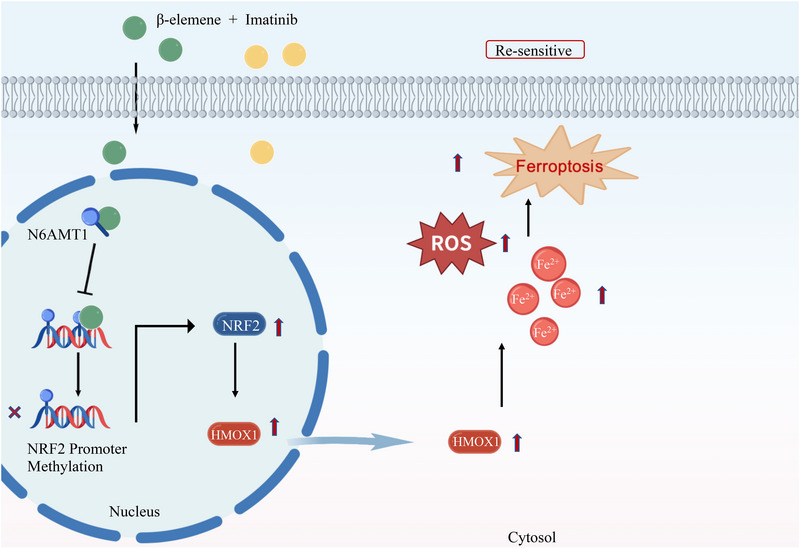
Summary diagram of the mechanism that β‐elemene increased the sensitivity of GIST cells to imatinib. β‐elemene specifically targets N6AMT1, inhibiting its transcriptional repression function and activating the NRF2‐HMOX1 signalling pathway to induce ferroptosis.

Imatinib resistance remains a thorny issue in the clinical setting of GIST. Hence, elucidation of the molecular mechanisms of imatinib resistance and developing a potential therapy strategy is pivotal. Many possible causes underlying imatinib resistance in GIST are widely acknowledged, including the emergence of secondary mutations in KIT kinase domains, unknown effects of gene polymorphisms, activation of alternative cell death pathways and so on.^6^, ^7^, ^28^ Our findings support the concept that ferroptosis plays an important role in imatinib resistance. Using GSEA, we found that ferroptosis activity is suppressed in imatinib‐resistant GIST and targeting ferroptosis could be a potential therapeutic option for GIST to eliminate tumours. However, many ferroptosis agonists are not currently in clinical use in GIST, and some phytochemicals are ideal adjuvants for therapy, which may have the potential to reverse the imatinib‐resistance of GIST cells. Here, we first showed that β‐elemene and imatinib synergistically induce ferroptosis in imatinib‐resistant GIST cells and validated this effect in various models. Further research shows that β‐elemene induced ferroptosis by promotion of Fe^2+^ accumulation and ROS to promote the antitumour effect. Mechanistically, β‐elemene binds to N6AMT1, which may inhibit the binding of N6AMT1 to NRF2 promoter regions, thereby activating the NRF2/HMOX1 pathway to induce ferroptosis (Figure 8).

Previous studies suggest that alterations to ferroptosis‐related signalling pathways may provide new insights into reversing treatment resistance.^42^, ^43^, ^44^ Therefore, drugs targeting ferroptosis in GIST cells hold promise as a prospective treatment strategy for patients with imatinib‐resistant GIST. In this study, we found the levels of Fe^2+^, ROS and lipid peroxidation were significantly elevated in cells treated with the combination of β‐elemene and imatinib. Besides, combination treatment inhibits the viability of GIST cells and promotes the sensitivity of imatinib in imatinib‐resistant GIST cells. Further, combination treatment‐mediated cell death was blocked by the ferroptosis inhibitor Fer‐1 (Figure 3D), indicating that β‐elemene synergy with imatinib effectively activates ferroptosis in imatinib‐resistant GIST cells. Subsequent experiments revealed that cotreatment with β‐elemene and imatinib induced the expression of HMOX1 to activate ferroptosis in GIST cells.

HMOX1, as a rate‐limiting enzyme in heme catabolism, can decompose heme into CO, Fe^2+^ and biliverdin, which play dual roles in ferroptosis. Several reports have revealed that the role of the NRF2/HOMX1 axis in ferroptosis is controversial. On one hand, it antagonises ferroptosis by inhibiting oxidation, and on the other hand, highly induced HMOX1 can aggravate heme degradation and release large amounts of free Fe^2+^. The excessive production of Fe^2+^ and ROS can promote ferroptosis.^45^, ^46^, ^47^ Thus, it is necessary to further elucidate the exact role of NRF2/HMOX1 induction in the β‐elemene‐mediated ferroptosis model. In the present study, high levels of HMOX1 were induced by the combination treatment of β‐elemene and imatinib. Furthermore, ferroptosis cell death was blocked when HMOX1 was inhibited by ZnPP. Overexpression of HMOX1 and hemin can promote ferroptosis cell death and imatinib sensitivity. Collectively, HMOX1 plays a pro‐ferroptosis role in the β‐elemene‐mediated ferroptosis model. And transfection of Nrf2 siRNA inhibited the expression of HMOX1 and reversed the ferroptosis caused by β‐elemene and imatinib treatment. Therefore, it is feasible to demonstrate that the NRF2/HMOX1 pathway plays a critical role in β‐elemene‐mediated GIST cell ferroptosis.

Accumulating evidence suggests that epigenetic regulation modulates the expression of ferroptosis‐associated genes.^48^, ^49^ DNA methylation is the most common epigenetic modification that has been studied in gene regulation. CDH1 promoter hypermethylation in head and neck cancer cells can suppress E‐cadherin expression and increase ferroptosis susceptibility.^50^ TPP assay has facilitated our identification of N6AMT1 as a binding target of β‐elemene. Molecular docking and CETSA further revealed that β‐elemene interacts with N6AMT1. N6AMT1 has been reported as a putative N_6_‐adenine‐specific DNA methyltransferase.^51^ High levels of N6AMT 1 are related to various forms of cancer, and siRNA directed at N6AMT1 can prevent the proliferation of cancer cells. Additional protein methylation substrates have also been identified for N6AMT1, which undergoes lysine monomethylation affecting transcription of cell cycle and cancer‐related genes,^52^ as well as methylation of translation‐related factors.^53^, ^54^ In our study, we found that β‐elemene may target N6AMT1 to inhibit DNA methylation in GIST cells. Pharmacologically targeting N6AMT1 with β‐elemene to disrupt N6AMT1 interaction with NRF2 promoter thereby leads to NRF2 and HMOX1 overexpression and lipid peroxide accumulation. Experiments from N6AMT1 knockdown by siRNA in vitro, and overexpression of N6AMT1, confirmed the hypothesis that N6AMT1 regulates the NRF2/HMOX1 pathway in GIST cells, pointing to a vital role of N6AMT1 in β‐elemene‐mediated ferroptosis.

## 5 CONCLUSION

In summary, our study showed that β‐elemene can improve Fe^2+^ level, MDA production and lipid peroxidation both in vivo and in vitro by activating the NRF2/HMOX1 pathway in GIST cells. N6AMT1 is a novel target protein of β‐elemene that contributes to its pro‐ferroptosis action by increasing NRF2 and HMOX1 expression. Our findings may indicate that β‐elemene as a prospective therapeutic strategy to improve the sensitivity of imatinib in GISTs.

## AUTHOR CONTRIBUTIONS

Xiaojun Wu and Weihao Li conceived and designed the study. Jin Lan, Weili Zhang, Kaixuan Zeng and Cong Li performed the main experiments. Jiahua He, Xinyue Li and Rong Yang performed bioinformatics analysis. Jun Chi, Zhigang Hong and Weifeng Wang analysed the data. Chi Zhou and Binyi Xiao conducted the formal analysis. Wenhua Fan, Junzhong Lin and Qingjian Ou interpreted the results. Yujing Fang perform software. Jin Lan wrote the manuscript. Jianhong Peng and Weihao Li wrote, reviewed and edited the paper. Zhizhong Pan, Jianhong Peng, Weihao Li and Xiaojun Wu supervised and acquired funding. All authors read and approved the final version of the manuscript.

## CONFLICT OF INTEREST STATEMENT

The authors declare no conflicts of interest.

## ETHICS STATEMENT

The present study was performed according to the ethical standards of the World Medical Association Declaration of Helsinki and was approved by the Institutional Review Board and Independent Ethics Committees of Sun Yat‐sen University Cancer Center. The informed consent requirement was waived by the ethics committees based on the nature of this retrospective study, in which patient data were kept confidential.

## Supporting information



Supporting Information

Supporting Information

Supporting Information

Supporting Information

Supporting Information

Supporting Information

Supporting Information

Supporting Information

Supporting Information

Supporting Information

Supporting Information

Supporting Information

Supporting Information

Supporting Information

## Data Availability

The datasets used and analysed during the current study are available from the corresponding author on reasonable request. The authenticity of this article has been validated by uploading the key raw data onto the Research Data Deposit public platform (www.researchdata.org.cn).
