# Supplementary figures and images for: β‐elemene promotes ferroptosis to improve the sensitivity of imatinib in gastrointestinal stromal tumours by targeting N6AMT1
Source: Clin Transl Med. 2025 Aug 27;15(9):e70438. doi: 10.1002/ctm2.70438 (PMC12390768; doi:10.1002/ctm2.70438)

A

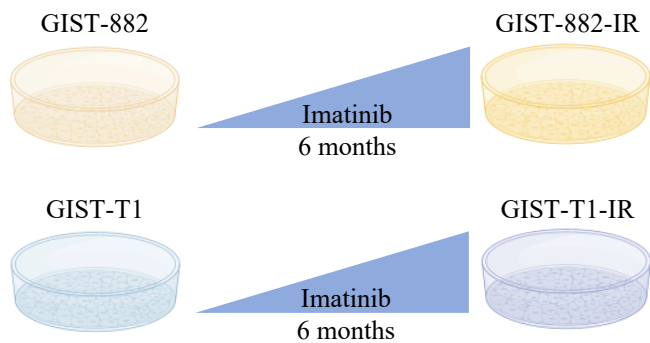

B

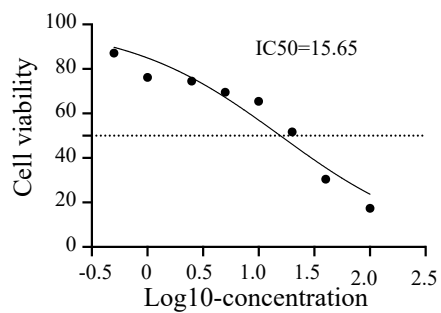

C

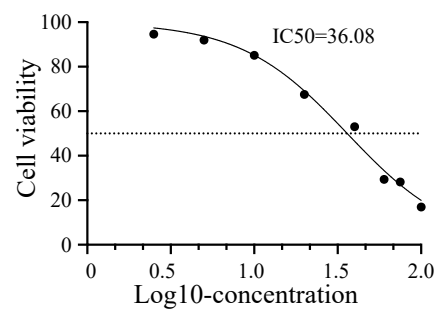

D

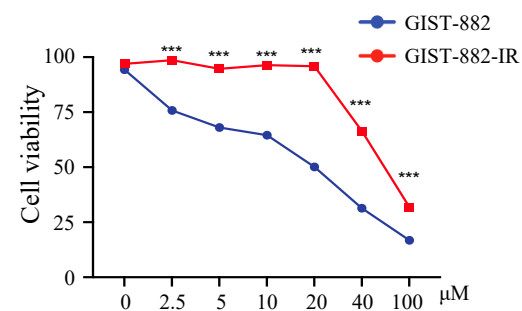

E

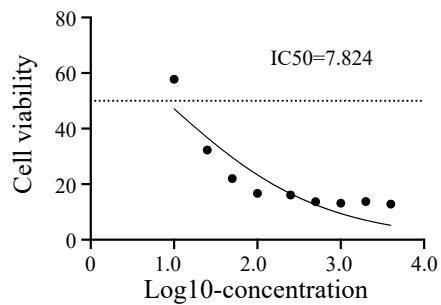

F

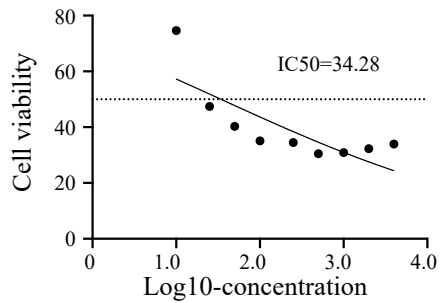

G

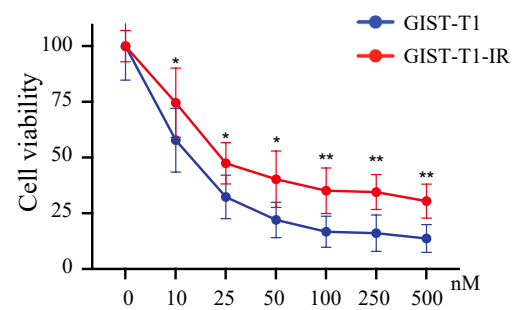

Supplement: Supplementary file 1 — Supporting Information [file CTM2-15-e70438-s001.pdf]

A

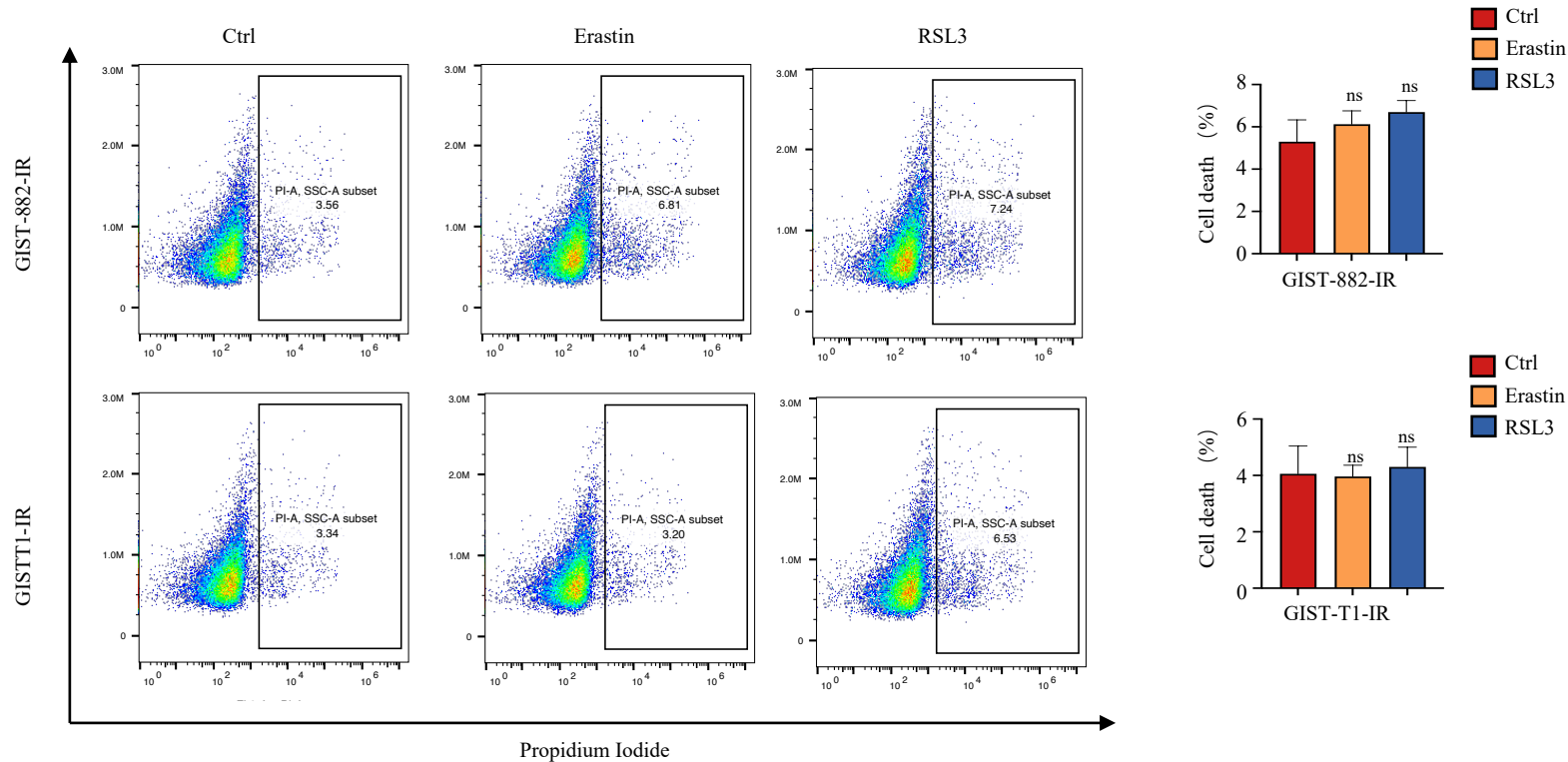

Supplement: Supplementary file 2 — Supporting Information [file CTM2-15-e70438-s003.pdf]

A

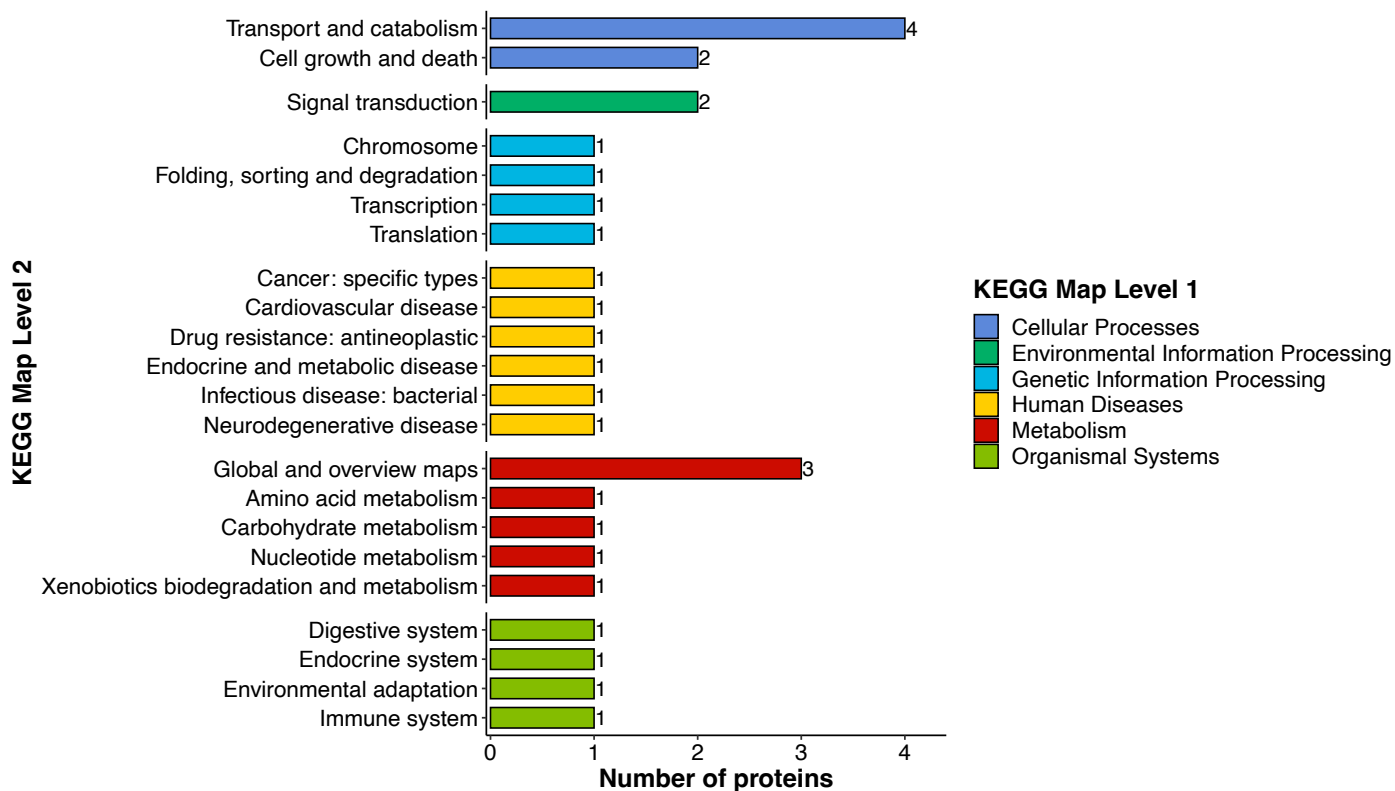

B

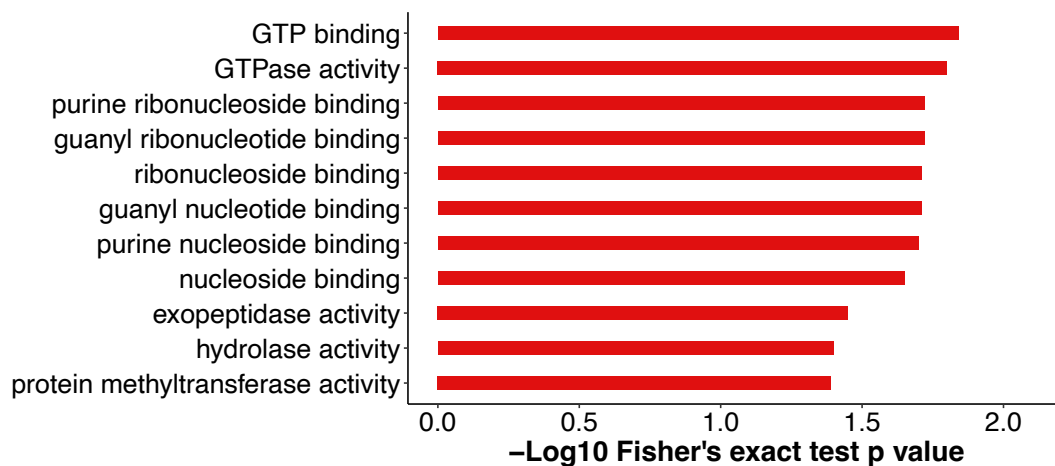

Supplement: Supplementary file 3 — Supporting Information [file CTM2-15-e70438-s005.pdf]

A

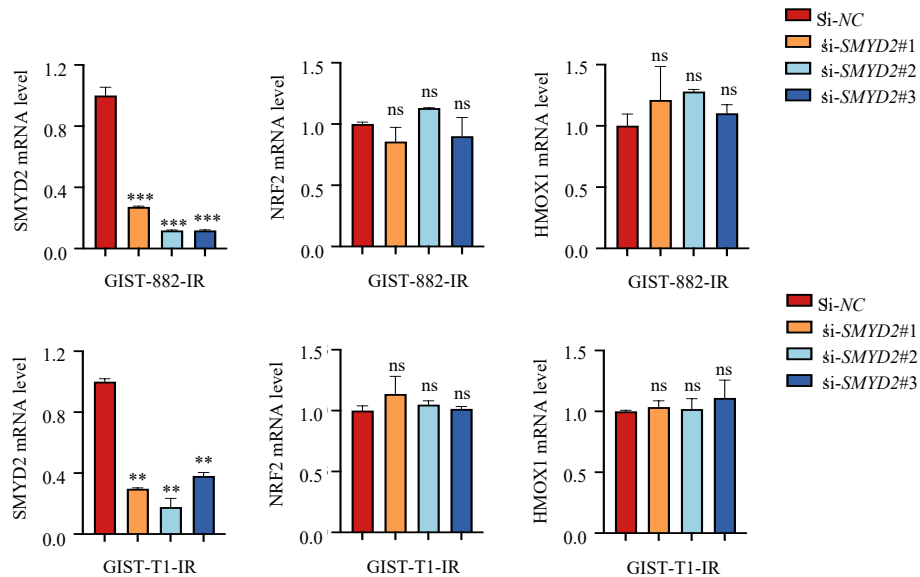

B

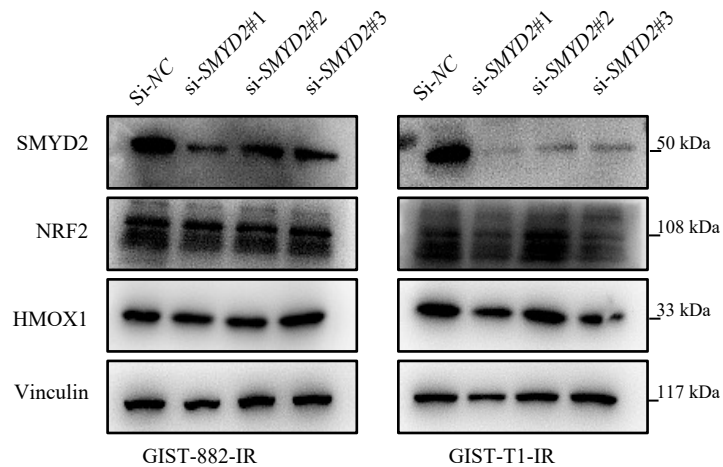

Supplement: Supplementary file 4 — Supporting Information [file CTM2-15-e70438-s012.pdf]

A

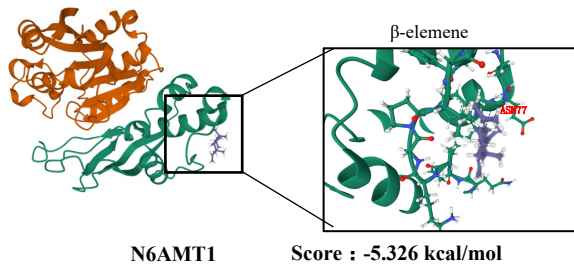

B

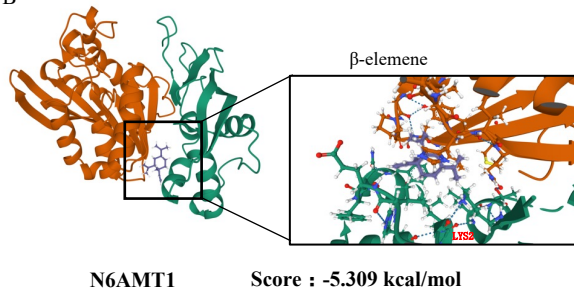

C

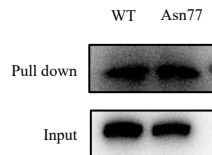

D

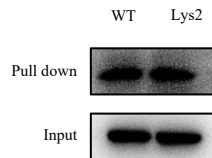

Supplement: Supplementary file 5 — Supporting Information [file CTM2-15-e70438-s010.pdf]

A

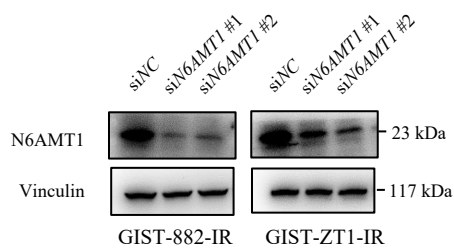

B

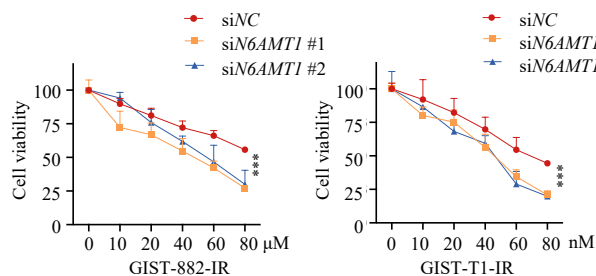

C

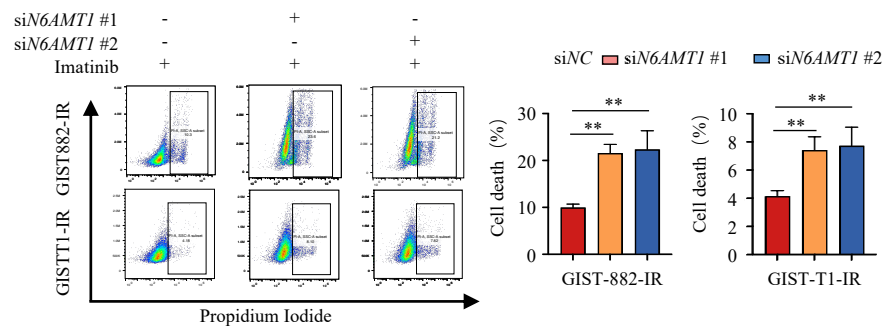

D

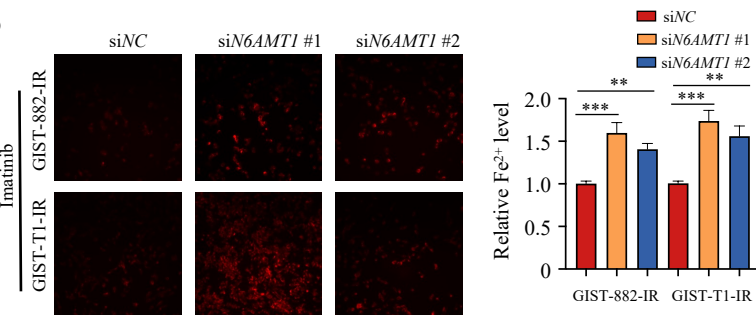

E

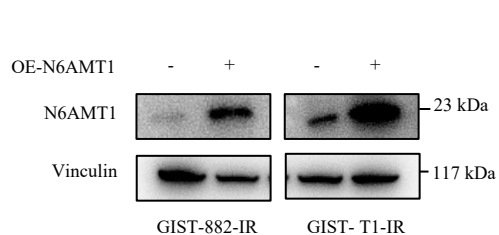

E

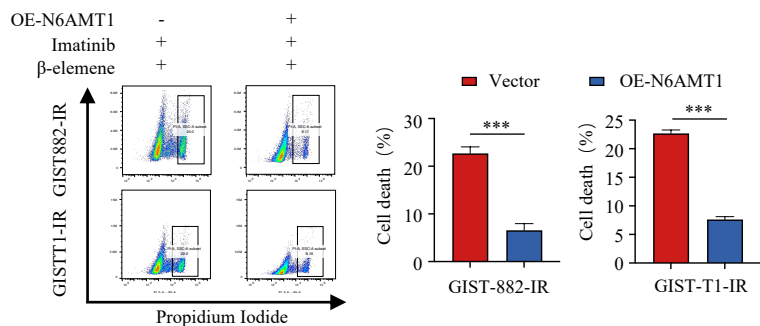

G

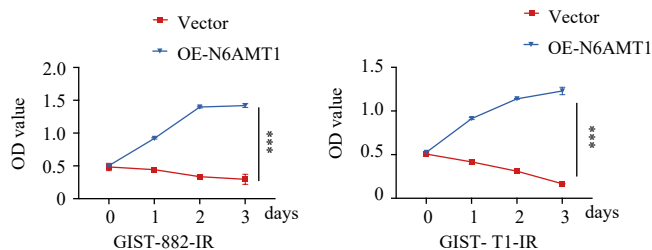

H

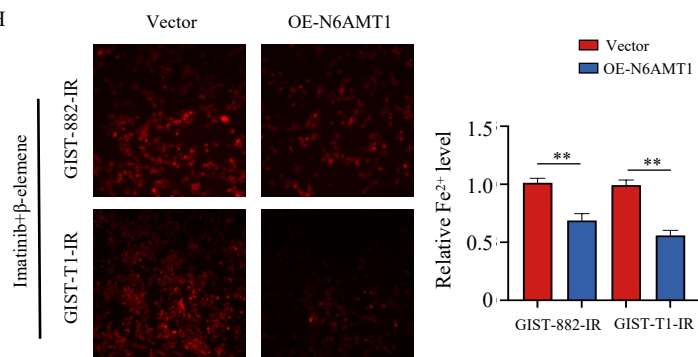

Supplement: Supplementary file 6 — Supporting Information [file CTM2-15-e70438-s009.pdf]

A

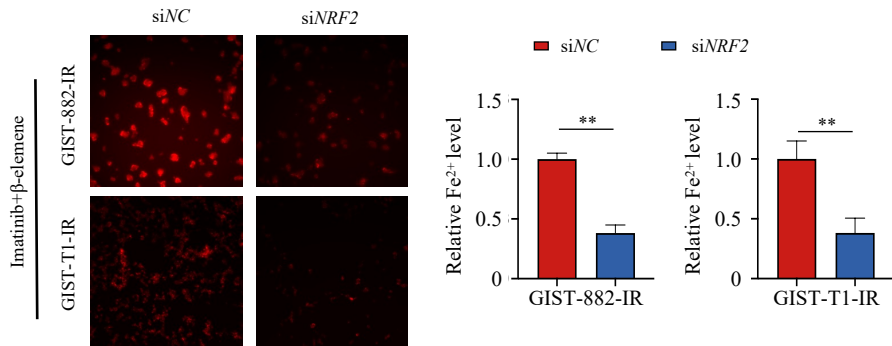

B

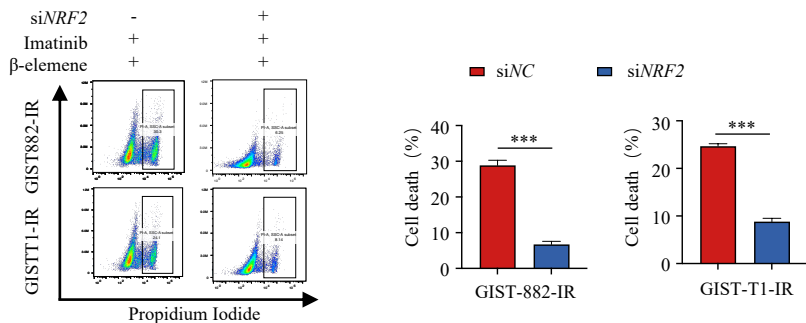

Supplement: Supplementary file 7 — Supporting Information [file CTM2-15-e70438-s013.pdf]

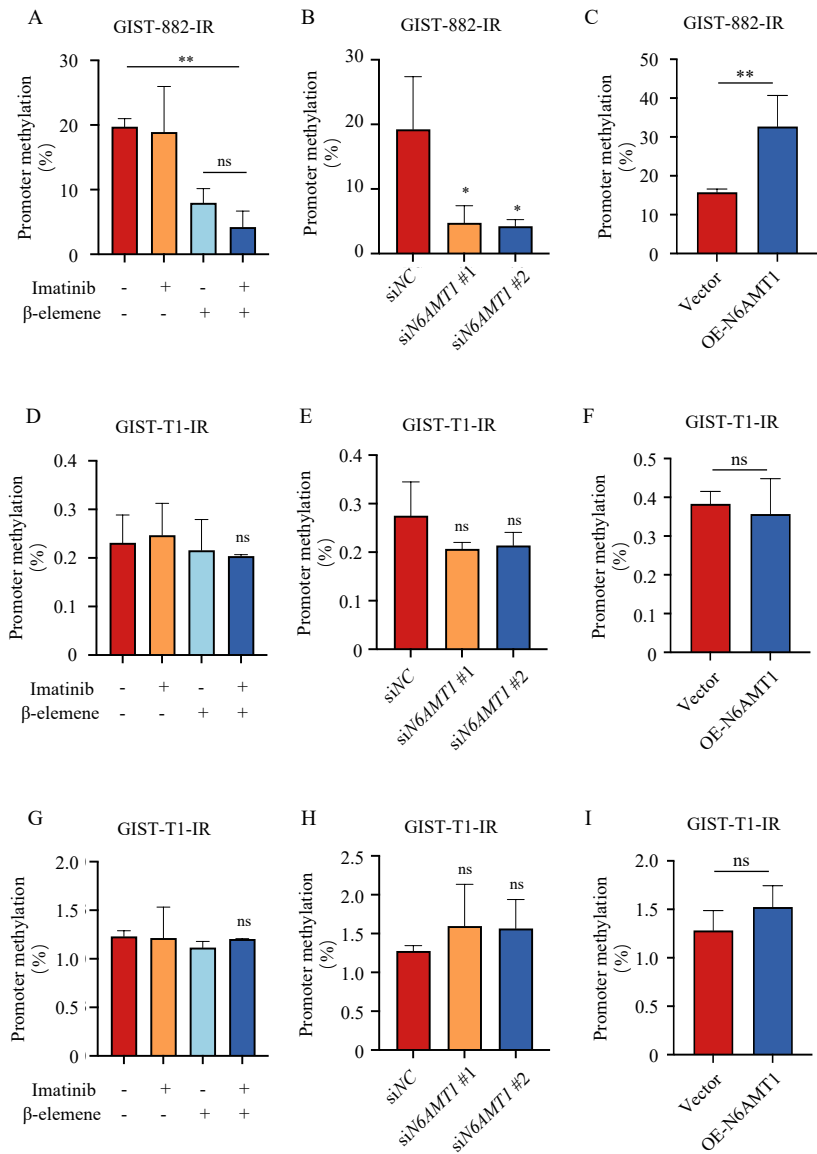

Supplement: Supplementary file 8 — Supporting Information [file CTM2-15-e70438-s006.pdf]

A

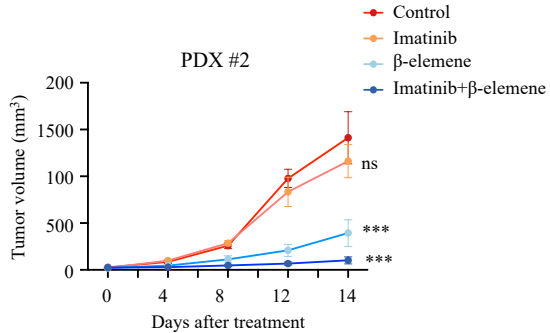

B

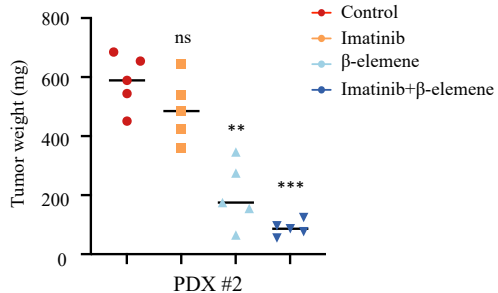

Supplement: Supplementary file 9 — Supporting Information [file CTM2-15-e70438-s002.pdf]

A

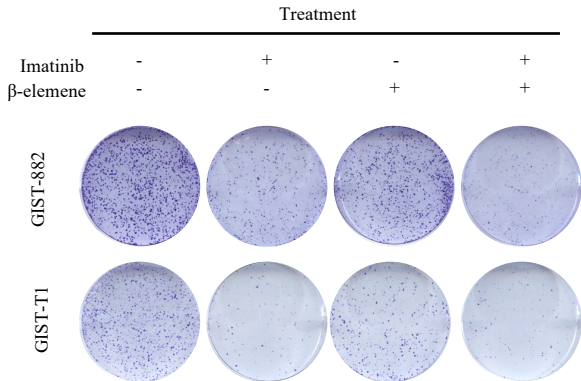

B

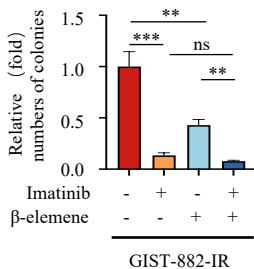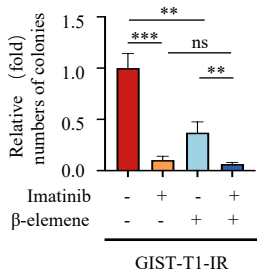

Supplement: Supplementary file 10 — Supporting Information [file CTM2-15-e70438-s007.pdf]
