# Supplementary material for: β‐elemene promotes ferroptosis to improve the sensitivity of imatinib in gastrointestinal stromal tumours by targeting N6AMT1
Source: Clin Transl Med. 2025 Aug 27;15(9):e70438. doi: 10.1002/ctm2.70438 (PMC12390768; doi:10.1002/ctm2.70438)
Supplement: Supplementary file 11 — Supporting Information [file CTM2-15-e70438-s004.docx]

**Table S2.** siRNA sequences used in this study.

| siRNA | Sense (5'-3') | Antisense (5'-3') |
| --- | --- | --- |
| siHO-1 | GGGUGAUAGAAGAGGCCAATT | UUGGCCUCUUCUAUCACCCTT |
| siN6AMT1 #1 | AGAAACUCUUUCAGUCCUCAA | UUGAGGACUGAAAGAGUUUCU |
| siN6AMT1 #2  siN6AMT1 #3  siSMYD2#1  siSMYD2#2  siSMYD2#3  siNRF2  siNC | GUUCACAUUCAACCAGUUAUU  CCUUUCACCAAGAGGAUUAUU  GGAAAGAAGGAUUGUCCAA  GAUAGAAAUGACCGGUUAA  GAUCAAACAGGAAAYYGAA  GGGAGGAGCUAUUAUCCAUTT UUCUCCGAACGUGUCACGUTT | AAUAACUGGUUGAAUGUGAAC  AAUAAUCCUCUUGGUGAAAGG  UUGGACAAUCCUUCUUUCC  UUAACCGGUCAUUUCUAUC  UUCAAUUUCCUGUUUGAUC  AUGGAUAAUAGCUCCUCCCTT ACGUGACACGUUCGGAGAATT |
