# Supplementary material for: β‐elemene promotes ferroptosis to improve the sensitivity of imatinib in gastrointestinal stromal tumours by targeting N6AMT1
Source: Clin Transl Med. 2025 Aug 27;15(9):e70438. doi: 10.1002/ctm2.70438 (PMC12390768; doi:10.1002/ctm2.70438)
Supplement: Supplementary file 12 — Supporting Information [file CTM2-15-e70438-s008.docx]

Table S3. Primer sequences for qPCR analysis.

| Genes (human) | Forward (5-3′) | Reverse (5-3′) |
| --- | --- | --- |
| GAPDH | GGCACCGTCAAGGCTGAGAAC | GGTGGCAGTGATGGCATGGAC |
| GPX4 | GAGGCAAGACCGAAGTAAACTAC | CCGAACTGGTTACACGGGAA |
| HMOX1 | AAGACTGCGTTCCTGCTCAACATC | CACGGTAAGGAAGCCAGCCAAG |
| NRF2 | ACGGTATGCAACAGGACATTGAGC | TGTGGAGAGGATGCTGCTGAAGG |
| FTH1 | AGAACTACCACCAGGACTCAGAGG | GGAAGATTCGGCCACCTCGTTG |
| NRF2 methylated | TTTTGTAATTTTAAATTAGGGAGGC | GAAAAACCTAAAAAAAATCTCCGTT |
| NRF2 unmethylated | TTGTAATTTTAAATTAGGGAGGTGT | AAAAAACCTAAAAAAAATCTCCATT |
| HMOX1 methylated | GAATGTGTTTGGAAGAGTGTTTTAC | CTATTTTACAAATAAAAAAACCGAA |
| HMOX1 unmethylated | ATGTGTTTGGAAGAGTGTTTTATGT | CTATTTTACAAATAAAAAAACCAAA |
| SMYD2  N6AMT1 | TACTGCAATGTGGAGTGTCAGA  GCAGGGGAGAACTTCGCTAC | ACAGTCTCCGAGGGATTCCAG  CAGCGCGTTCAAAAGCAGAAA |
