# Supplementary material for: β‐elemene promotes ferroptosis to improve the sensitivity of imatinib in gastrointestinal stromal tumours by targeting N6AMT1
Source: Clin Transl Med. 2025 Aug 27;15(9):e70438. doi: 10.1002/ctm2.70438 (PMC12390768; doi:10.1002/ctm2.70438)
Supplement: Supplementary file 13 — Supporting Information [file CTM2-15-e70438-s011.docx]

Table S1. Possible protein targets of β-elemene identified by TPP.

| **Gene Name** | **Protein Description** |
| --- | --- |
| CNOT1  MIEF1  TRIM38  IMPA2  SCAMP2  TOX3  SYNGR2  TBC1D4  CTSV  PTCD1  SUN1  PTTG1  CD63  TRAPPC2  TOP2A  ITIH2  KRT2  ANXA11  RAB10  LYZ  TBC1D8B  TAF13  RABL3  BRMS1L  INTS5  PASD1  CDAN1  CHMP7  RNF141  SMPDL3B  DPF1  TUBGCP5  MTG1  UCK2  RAB1B  SMYD2  ANKRD10  MSRB1  VPS54  GGA2  MAGED2  MFHAS1  POLR3H  N6AMT1  CPQ  ZHX2  MRPL33  COMMD2  LDAH  TRANK1  ANKRD52  TOR4A | CCR4-NOT transcription complex subunit 1  Mitochondrial Elongation Factor 1  E3 ubiquitin-protein ligase TRIM38  Inositol monophosphatase 2  Secretory carrier-associated membrane protein 2  TOX high mobility group box family member 3  Synaptogyrin-2  TBC1 domain family member 4  Cathepsin L2  Pentatricopeptide repeat-containing protein 1  SUN domain-containing protein 1  Securin  CD63 antigen  Trafficking protein particle complex subunit 2  DNA topoisomerase 2-alpha  Inter-alpha-trypsin inhibitor heavy chain H2  Keratin, type II cytoskeletal 2 epidermal  Annexin A11  Ras-related protein Rab-10  Lysozyme C  TBC1 domain family member 8B  Transcription initiation factor TFIID subunit 13  Rab-like protein 3  Breast cancer metastasis-suppressor 1-like protein  Integrator complex subunit 5  Circadian clock protein PASD1  Codanin-1  Charged multivesicular body protein 7  RING finger protein 141  Acid sphingomyelinase-like phosphodiesterase 3b  Zinc finger protein neuro-d4  Gamma-tubulin complex component 5  Mitochondrial ribosome-associated GTPase 1  Uridine-cytidine kinase 2  Ras-related protein Rab-1B  N-lysine methyltransferase SMYD2  Ankyrin repeat domain-containing protein 10  Methionine-R-sulfoxide reductase B1  Vacuolar protein sorting-associated protein 54  ADP-ribosylation factor-binding protein GGA2  Melanoma-associated antigen D2  Malignant fibrous histiocytoma-amplified sequence 1  DNA-directed RNA polymerase III subunit RPC8  Methyltransferase N6AMT1  Carboxypeptidase Q  Zinc fingers and homeoboxes protein 2  Large ribosomal subunit protein bL33m  COMM domain-containing protein 2  Lipid droplet-associated hydrolase  TPR and ankyrin repeat-containing protein 1  Serine/threonine-protein phosphatase 6 regulatory ankyrin repeat subunit C  Torsin-4A |
| RBM18 | Probable RNA-binding protein 18 |
