# Supplementary material for: β‐elemene promotes ferroptosis to improve the sensitivity of imatinib in gastrointestinal stromal tumours by targeting N6AMT1
Source: Clin Transl Med. 2025 Aug 27;15(9):e70438. doi: 10.1002/ctm2.70438 (PMC12390768; doi:10.1002/ctm2.70438)
Supplement: Supplementary file 14 — Supporting Information [file CTM2-15-e70438-s014.docx]

Table S4.. Clinical characteristics data of β-elemene treatment cohort:

| No. | Gender | Age | β-elemene treatment evaluation | Progression Free Survival (Month) |
| --- | --- | --- | --- | --- |
| 1 | Male | 53 | SD | 11.4 |
| 2 | Male | 62 | SD | 11.2 |
| 3 | Female | 57 | PR | 5 |
| 4 | Female | 39 | SD | 8.8 |
| 5 | Male | 69 | SD | 3.5 |
| 6  8 | Female  Female | 54  67 | PD  SD | /  4.2 |
